# Ciprofloxacin-induced microbiota dysbiosis triggers seizure susceptibility through the microbiota-gut-brain axis

**DOI:** 10.3389/fimmu.2026.1670694

**Published:** 2026-03-31

**Authors:** Qihang Zou, Yaqian Zhang, Shangnan Zou, Yinchao Li, Huifeng Li, Man Yang, Huanling Lai, Ruili Niu, Xiaofeng Yang, Liemin Zhou

**Affiliations:** 1 Clinical Neuroscience Center, The Seventh Affiliated Hospital of Sun Yat-sen University, Shenzhen, Guangdong, China; 2 Department of Rehabilitation Medicine, The Third Affiliated Hospital of Southern Medical University, Guangzhou, China; 3 Sun Yat-sen Memorial Hospital, Sun Yat-sen University, Guangzhou, China; 4 Guangzhou Laboratory, Guangzhou, Guangdong, China; 5 Department of neurology, The First Affiliated Hospital, Sun Yat-sen University, Guangzhou, China

**Keywords:** microbiota dysbiosis, seizure susceptibility, intestinal and blood-brain barriers, gut-brain axis, neuroinflammation

## Abstract

**Background:**

Epilepsy is linked to inflammation and gut microbiota dysbiosis. Ciprofloxacin-induced microbiota disruption may increase seizure susceptibility. This study investigates underlying mechanisms and the therapeutic potential of fecal microbiota transplantation (FMT).

**Methods:**

A total of 64 male Sprague-Dawley rats were categorized into four experimental groups: Control (CTRL), Ciprofloxacin-treated (CPF), CPF with fecal microbiota transplantation (CPF-FMT), and CPF with phosphate-buffered saline (CPF-PBS). Gut microbiota dysbiosis was induced with ciprofloxacin for 14 days, followed by either FMT or PBS for 14 days. Seizure susceptibility was assessed using pentylenetetrazole (PTZ), alongside molecular analyses of gut and blood-brain barrier integrity, neuroinflammatory markers, and cortical transcriptomics.

**Results:**

Microbiota dysbiosis was associated with increased seizure susceptibility, accompanied by disruption of intestinal and blood-brain barrier (BBB) integrity, thereby exacerbating systemic and neuroinflammation. Dysbiotic rats exhibited significant reductions in microbial diversity and depletion of protective taxa, including f_Muribaculaceae, f_Prevotellaceae, and Lachnospiraceae_NK4A136_group, which correlated with intestinal barrier dysfunction. This dysfunction was associated with reduced tight junction proteins (ZO-1, Occludin, Claudin-5) and inflammatory cell infiltration. Systemic inflammation and disrupted blood-brain barrier integrity resulted in microglial activation and astrocytic proliferation in the brain. Notably, FMT was related to restoration of microbial diversity, improvement of barrier-related markers, attenuation of neuroinflammatory responses, and a reduction in seizure susceptibility.

**Conclusion:**

This study provides evidence linking gut microbiota dysbiosis to seizure susceptibility through neuroinflammatory processes, contributing to the understanding of gut–brain axis involvement in fluoroquinolone-induced seizures.

## 1 Introduction

Epilepsy is a common neurological condition impacting over 65 million individuals globally, and emerging evidence suggests that inflammation plays a crucial role in its pathogenesis (1, 2). Moreover, increasing research indicates that gut microbiota homeostasis can modulate immune responses via the gut-brain axis (3, 4). The gut-brain axis (GBA) serves as a bidirectional signaling pathway between the gut and the central nervous system (CNS) (5). The gut microbiota plays a key role in this interaction by modulating immune responses, producing neuroactive metabolites and maintaining barrier integrity (3, 6–8).

Microbiota dysbiosis is associated with the development and treatment of epilepsy (9–15). Changes in the composition and abundance of multiple microbial taxa have been reported in patients with epilepsy. Studies have shown that the abundance of Proteobacteria, Verrucomicrobia, Fusobacteria, and Firmicutes increased, and the abundance of Actinobacteria and Bacteroidetes decreased in epilepsy (16–19). Following lithium-pilocarpine-induced status epilepticus (SE), alterations in the gut microbiota have been observed in epileptic rats (20). Notably, some studies suggest that the therapeutic effects of the ketogenic diet on epilepsy may be mediated through gut microbiota modulation (9). In addition, the use of probiotic preparations has been shown to attenuate seizures in animal studies and clinical trials (9, 21–24). Gut microbiota plays a crucial role in regulating neuroinflammation and seizure susceptibility. External factors such as antibiotics can disrupt microbial balance, potentially leading to significant neurological consequences. Previous studies have shown that targeting the gastrointestinal tract with the antibiotic rifaximin reduces seizure duration in TLE mice (25). However, the clinical application of antibiotics does not always yield beneficial health effects, and their impact on the gut microbiota in patients remains to be fully elucidated. Notably, fluoroquinolone antibiotics have been reported to induce drug-associated seizures in patients without a prior history of epilepsy (26, 27). To investigate whether fluoroquinolones influence seizure susceptibility through gut microbiota alterations, our previous study demonstrated that inducing gut microbiota dysbiosis in rats with the third-generation fluoroquinolone ciprofloxacin increased seizure susceptibility (15). Nevertheless, the molecular mechanisms underlying the relationship between ciprofloxacin-induced gut microbiota dysbiosis and seizure susceptibility require further investigation.

Based on the above evidence, this study aimed to investigate the molecular mechanisms underlying ciprofloxacin-induced microbiota dysbiosis and its impact on increased seizure susceptibility. To assess seizure susceptibility, we employed a pentylenetetrazole (PTZ)-induced seizure model in rats. Specifically, we examined whether ciprofloxacin administration altered gut microbiota composition and intestinal structure, and we evaluated its effects on inflammation and blood-brain barrier integrity. Furthermore, we demonstrate the therapeutic potential of fecal microbiota transplantation (FMT) in reversing these pathological changes, providing new insights into microbiota interventions targeting seizures.

## 2 Materials and methods

### 2.1 Experimental animals and grouping

Sprague-Dawley rats (5–6 weeks old, 180 ± 20 g) were purchased from Beijing Vital River Laboratory Animal Technology Co., Ltd. The Institutional Ethical Committee for Animal Welfare of the Seventh Affiliated Hospital of Sun Yat-sen University(approval No. SYSU-IACUC-2022-B1339) approved the study. Rats were housed in a temperature-controlled room with a 12-h light/dark cycle under specific pathogen–free (SPF) conditions, with free access to food and water.

Rats were randomly assigned to two groups: Control (CTRL, n = 22) and Ciprofloxacin-treated (CPF, n = 42). Among the CTRL rats, 8 were designated as microbiota donors (CTRL-PBS, n = 8). To induce gut microbiota dysbiosis, CPF rats received ciprofloxacin monohydrochloride (TargetMol, USA; 10 mg/mL in sterile saline, Shuanghe Pharmaceutical Co., China) at 100 mg/kg via oral gavage for 14 consecutive days. CTRL and CTRL-PBS rats were gavaged with equivalent volumes of sterile saline. From Days 15 to 28, CTRL-PBS rats received PBS (Meilunbio, China) by oral gavage every other day and served as healthy microbiota donors for FMT in CPF-FMT rats.

After the 14-day ciprofloxacin treatment, 8 rats each from the CPF and CTRL groups underwent PTZ-induced seizure testing, while the remaining 6 rats per group were sacrificed for molecular and histological analyses of colonic and cortical tissues. The 28 remaining CPF rats were divided into two subgroups: CPF-FMT (n = 14) and CPF-PBS (n = 14). CPF-FMT rats received FMT every other day from Days 15 to 28 using Fecal microbial solution from CTRL-PBS donors, while CPF-PBS rats received PBS as a placebo control. After the intervention, 8 rats from each subgroup underwent PTZ-induced seizure testing, and the remaining 6 were sacrificed for analyses of the integrity of the colon and blood-brain barrier and neuroinflammatory markers in cortical tissues.

### 2.2 Induction of gut microbiota dysbiosis

Gut microbiota dysbiosis was induced by oral gavage of ciprofloxacin monohydrochloride (TargetMol, USA; 10 mg/mL dissolved in sterile saline) at a dosage of 100 mg/kg body weight once daily for 14 consecutive days. Control rats were administered an equivalent volume of sterile saline (10 mL/kg) via oral gavage over the same period.

### 2.3 FMT

FMT was performed to reconstitute gut microbiota composition (28, 29). Fresh feces from healthy donor rats (CTRL-PBS group) were suspended in PBS (1 g feces/5 mL PBS), centrifuged at 2500 rpm for 10 min at 4 °C, and filtered through a nylon mesh (40-μm) to prepare the microbial suspension. CPF-FMT rats received 10 mL/kg of this suspension daily via oral gavage for 14 days, starting on Day 15 after ciprofloxacin treatment. CPF-PBS rats were gavaged with an equivalent volume of PBS as a control.

### 2.4 PTZ-induced seizure susceptibility testing

On Day 14 and Day 28 of the experiment, rats were implanted with cranial electrodes and allowed to recover for two days. And then PTZ (45 mg/kg) was administered intraperitoneally to induce seizures. Seizure activity was monitored using synchronized video-electroencephalography (VEEG) with LabChart software. Baseline EEG was recorded for 10 minutes before PTZ injection, followed by continuous recording for 90 minutes post-injection. Seizure severity was evaluated using the modified Racine scale: Score 0: No response; Score I: Rhythmic facial or mouth movements; Score II: Head nodding or tail jerking; Score III: Clonic movements of one limb; Score IV: Bilateral limb clonic movements or tonic extension; Score V: Loss of balance and falling.

### 2.5 16S rRNA sequencing and analysis

Fecal samples were gathered on Day 0 (baseline), Day 14 (post ciprofloxacin), and Day 28 (post FMT or PBS treatment). 16S rRNA sequencing was conducted by Guangdong Magigene Biotechnology Co., Ltd.

### 2.6 Histological and immunohistochemical analysis

Histological evaluation of colonic tissues was conducted using hematoxylin and eosin (H&E) staining. Immunohistochemical staining was performed to detect tight junction proteins, including ZO-1 (1:2000, 21773-1-AP, Proteintech) and Occludin (1:8000, 27260-1-AP, Proteintech), in colonic and brain tissues. Additionally, glial fibrillary acidic protein (GFAP; 1:2000, 80788, Cell Signaling Technology) staining was performed on brain tissues. Quantification of staining was carried out using 3–5 representative fields per sample.

### 2.7 Western blot analysis

Tight junction proteins (ZO-1, Occludin, and Claudin-5) in intestinal and brain tissues were assessed using Western blotting. Tissues were homogenized in RIPA lysis buffer (P0013B, Beyotime, China) with the addition of protease and phosphatase inhibitors (P1005, Beyotime, China; P1045, Beyotime, China). Enhanced BCA Protein Assay Kit (P0010S, Beyotime, China) was used to determine protein concentrations. Equal quantities of protein were separated via SDS-PAGE, transferred to NC membranes, and then blocked. Membranes were incubated with primary antibodies against ZO-1 (1:5000, 21773-1-AP, Proteintech), Occludin (1:8000, 27260-1-AP, Proteintech), Claudin-5 (1:5000, 29767-1-AP, Proteintech), and GAPDH (1:10000, 60004-1-Ig, Proteintech), followed by HRP-conjugated secondary antibodies (anti-rabbit IgG, 1:1000, A0208, Beyotime; anti-mouse IgG, 1:5000, ab205719, Abcam). Signals were visualized using chemiluminescence (Tanon 4800 Multi, China).

### 2.8 RNA sequencing analysis

Cortical tissues from the CPF and CTRL groups (n = 3 per group) were subjected to RNA sequencing, performed by Lianchuan Biotechnology Co. (Hangzhou, China). Total RNA was extracted, and transcriptomic libraries were prepared and sequenced. The R language was used to identify differentially expressed genes (DEGs) and conduct the KEGG and GO enrichment analysis, with significance thresholds set at P < 0.05 and |log2FC| > 2 for differential expression analysis. PPI network was established using STRING website, Cytoscape software was used to identify hub genes, and NetworkAnalyst was used for transcription factor prediction and interaction network construction. Immunity-related genes were obtained from The Immunology Database and Analysis Portal (ImmPort).

### 2.9 Immunofluorescence analysis

Microglial activation was evaluated using immunofluorescence co-staining of Iba1 with CD14 and CD68. Cortical sections were applied with primary antibodies against Iba1 (1:100, ab283319, Abcam), CD14 (1:100, 17000-1-AP, Proteintech), and CD68 (1:100, 28058-1-AP, Proteintech), Fluorescent dye-conjugated secondary antibodies (CoraLite488-conjugated Goat Anti-Rabbit IgG (H+L) were used in the next step, 1:1000, SA00013-2, Proteintech; Alexa Fluor^®^ 594-conjugated Goat Anti-Mouse IgG (H+L) F(ab’)_2_ Fragment, 1:1000, 8890, Cell Signaling Technology). Fluorescent images were captured using Mshot MF43-N. The number and percentage of Iba1+CD14+ and Iba1+CD68+ cells in cortical tissues were evaluated.

### 2.10 Cytokine analysis

Serum concentrations of lipopolysaccharide-binding protein (LBP), interleukin-6 (IL-6), and tumor necrosis factor-alpha (TNF-α) were determined in serum and cortex, using enzyme-linked immunosorbent assay (ELISA) kits(Cusabio, China). Due to the rapid *in vivo* fluctuations of lipopolysaccharide (LPS) and the technical challenges of its direct measurement, we used LBP as a surrogate marker to reflect LPS levels and immune burden. LBP is a recognized biomarker of LPS exposure and systemic inflammation (30).

### 2.11 Statistical analysis

Data were analyzed with SPSS software (version 25.0; IBM, Somers, NY, USA). Continuous data were expressed as mean ± SD, and categorical variables were summarized as frequencies or percentages. Normally distributed numerical data were performed Student’s *t*-test, non-normally distributed data were analyzed by the Mann–Whitney *U* test. Kaplan–Meier survival curves were constructed to evaluate seizure probability. The data analysis of 16S rRNA sequencing was conducted using the Magigene cloud platform (http://cloud.magigene.com). *P* < 0.05 was considered statistically significant.

## 3 Results

### 3.1 Gut microbiota dysbiosis enhances seizure susceptibility in rats

**Figure 1:**
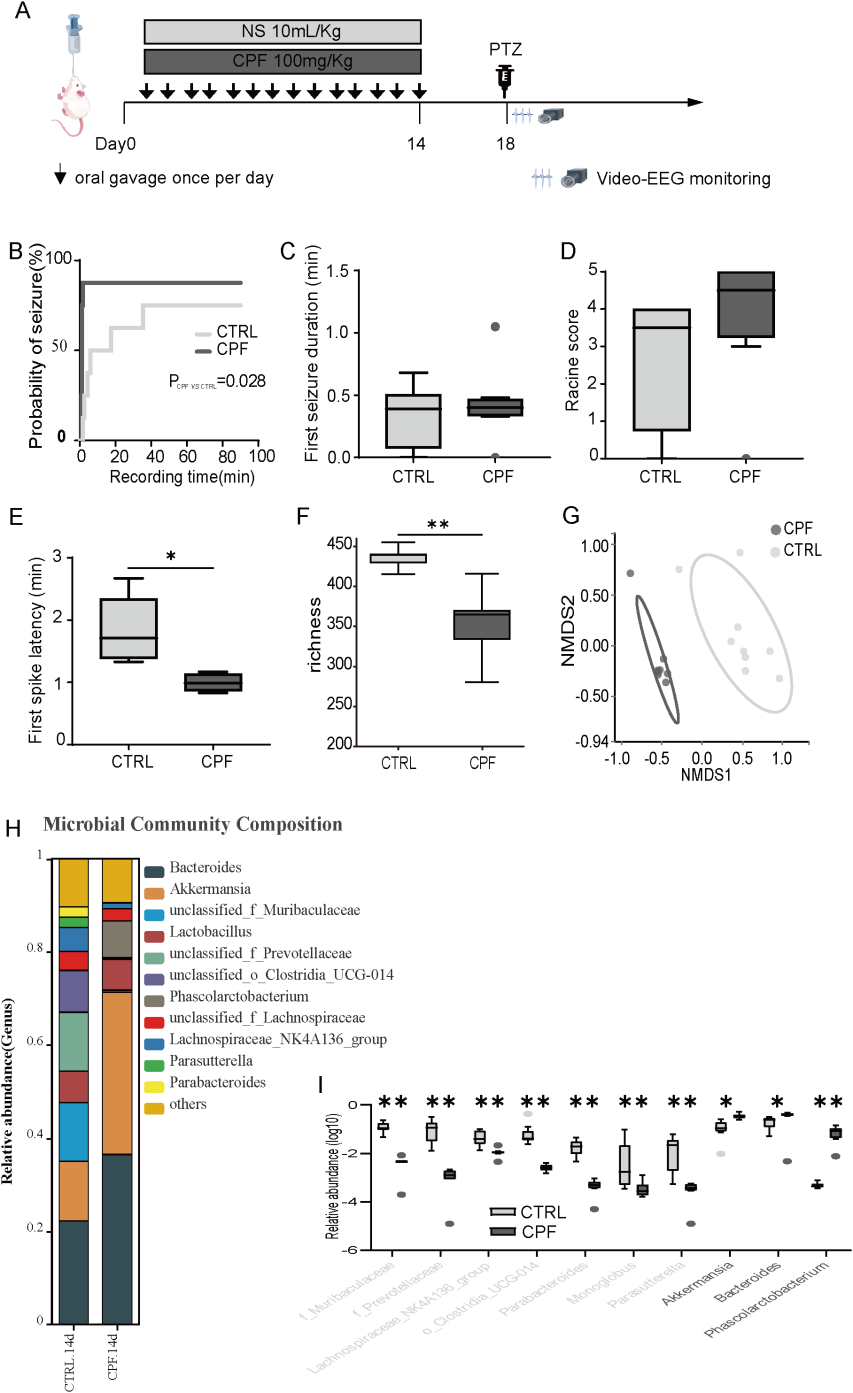
Gut microbiota dysbiosis enhances seizure susceptibility in rats. **(A)** Experimental timeline. **(B)** Kaplan-Meier survival curve for seizure latency. **(C)** Seizure duration of the first Racine score ≥3 seizures. **(D)** Median Racine stage. **(E)** Latency to first spike. **(F)** Alpha diversity (richness index) after 14 days of treatment. **(G)** NMDS plot showing distinct clustering of CPF and CTRL groups. **(H)** Microbial community composition. **(I)** Top 10 differentially abundant taxa enriched in CPF (dark gray) and CTRL (light gray) groups. **(A–E)**, n = 8 per group; **(F–I)**, CPF (n = 8) and CTRL (n = 9). Statistical comparisons were performed using the Mann-Whitney U test (*P < 0.05, **P < 0.01, ***P < 0.001, ****P < 0.0001).

An overview of the experimental design is shown in **Figure 1A**. Kaplan-Meier survival analysis revealed that seizure latency was significantly shorter in the CPF group than in the CTRL group (1.13 min vs. 35.35 min, Log-rank P = 0.028; **Figure 1B**). This indicates that gut microbiota dysbiosis increased seizure susceptibility. Similarly, the CPF group exhibited a higher median Racine stage (4.50; IQR: 3.25–5.00) than the CTRL group (3.50; IQR: 0.75–4.00), not significant (P = 0.08; **Figure 1D**). However, the duration of the first seizure showed no significant difference between these two groups (P = 0.792; **Figure 1C**). Additionally, the latency to the first spike was significantly shorter in the CPF group (0.99 min vs. 1.71 min, P = 0.012; **Figure 1E**). These results suggest that gut microbiota dysbiosis alters seizure severity and onset.

To explore the impact of ciprofloxacin on gut microbiota and its potential relevance to seizure susceptibility, we analyzed fecal samples collected at baseline (Day 0) and Day 14 using 16S rRNA sequencing. At baseline, the gut microbiota alpha and beta diversity did not differ significantly between the CPF and CTRL groups. (P > 0.05, **Supplementary Figures 1A, B**). The microbial community composition at the genus level was nearly identical between the CPF.0d and CTRL.0d groups, indicating consistent baseline levels before the experiment (**Supplementary Figure 1F**). However, after 14 days of ciprofloxacin administration, alpha diversity was markedly decreased in the CPF group relative to the CTRL group (P = 0.001, **Figure 1F**). NMDS analysis indicated a unique gut microbiota clustering pattern in the CPF group compared to the CTRL group (**Figure 1G**), indicating substantial microbiota disruption. Significant differences in microbial composition at the genus level were observed between the CTRL.14d and CPF.14d groups, reflecting the impact of ciprofloxacin on gut microbiota. Ciprofloxacin treatment significantly altered the abundance of key gut microbial taxa (**Figures 1H, I**). *Akkermansia* and *Bacteroides* were notably enriched in the CPF group, whereas several taxa, including f_Muribaculaceae, f_Prevotellaceae, Lachnospiraceae_NK4A136_group, o_Clostridia_UCG-014, and *Parasutterella*, *Parabacteroides*, were markedly depleted. The reduction of these protective taxa highlights a potential link between microbiota dysbiosis and increased seizure susceptibility.

### 3.2 Gut microbiota disruption modulated inflammation-related pathways in the brain affecting excitatory/inhibitory balance

**Figure 2:**
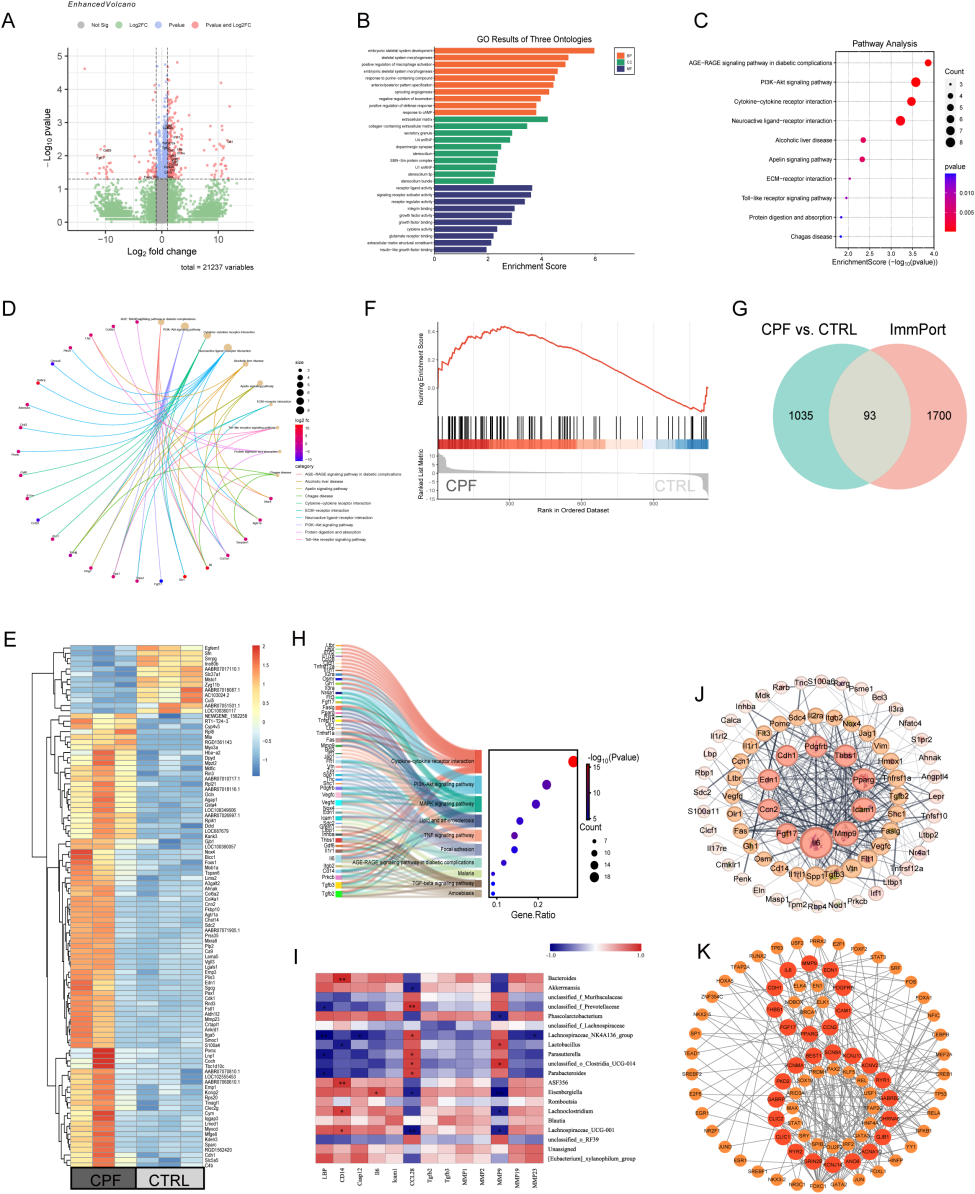
Inflammation-related pathways in brain. **(A)** Volcano plot of differentially expressed genes (DEGs) identified by RNA sequencing comparing CPF (n = 3) and CTRL (n = 3) groups, showing 138 DEGs (P < 0.05, |log2FC| > 2). **(B)** GO enrichment analysis of 138 DEGs. **(C)** KEGG pathway enrichment analysis of 138 DEGs. **(D)** Chord diagram showing the top 10 KEGG enrichment results. **(E)** Heatmap of the top 100 DEGs. **(F)** Gene Set Enrichment Analysis (GSEA) highlighting significant enrichment of inflammation-related gene sets in the CPF group. **(G)** Venn diagram of DEGs in gut microbiota dysbiosis rats and immunity-related genes obtained from the Immunology Database and Analysis Portal (ImmPort). **(H)** KEGG pathway enrichment analysis of 93 inflammation-related DEGs. **(I)** Microbial community and inflammation-related gene correlation heatmap analysis. **(J)** Protein-protein interaction (PPI) network of 93 inflammation-related genes. **(K)** Predicted transcription factor interaction network of 10 inflammation-related hub genes and ion channel genes with differential expression in gut microbiota dysbiosis rats.

To explore the mechanism of gut microbiota dysbiosis affecting seizure susceptibility through central inflammatory and neuromodulatory mechanisms, we performed RNA sequencing of the cerebral cortex transcriptome of the CTRL and CPF groups. 1035 differentially expressed genes (DEGs, P < 0.05) were identified, of which 138 genes met the higher significance criterion (P < 0.05, |log2FC| > 2) (**Figure 2A**). Among the differentially expressed genes, LBP encodes a protein that binds to the bacterial outer membrane antigen LPS. Compared to the CTRL group, LBP was significantly upregulated in the CPF group. Additionally, the downstream inflammatory cytokine IL-6 exhibited increased expression in the CPF group. KEGG analysis revealed significant enrichment of these genes in the PI3K-Akt signaling pathway, cytokine-cytokine receptor interaction, ECM-receptor interaction, and Toll-like receptor (TLR) signaling pathways (**Figures 2B–D**). Heat map and GSEA analysis further revealed that pro-inflammation-related genes (including LBP, CD14, IL-6) were significantly up-regulated in the CPF group (**Figures 2E, F**), supporting the activation of central inflammation. In addition, cross-matching the DEGs with the ImmPort database revealed 93 overlapping genes (**Figure 2G**), which were significantly enriched in the MAPK signaling pathway (**Figure 2H**), suggesting that the TLR pathway may contribute to increased seizure susceptibility induced by gut microbiota dysbiosis through activation of downstream MAPK signaling. We created a heatmap showing the correlation between inflammation-related genes and gut microbiota (**Figure 2I**). The results showed that f_Prevotellaceae, Lachnospiraceae_NK4A136_group, *Lactobacillus*, *Parasutterella*, o_Clostridia_UCG-014 and *Parabacteroides* were associated with LBP, CD14, Casp12, and IL-6 gene expression negatively, and positively with CCL28, a gene that regulates mucosal immunity. In contrast, *Bacteroides*, ASF356, *Eisenbergiella*, *Lachnoclostridium*, and Lachnospiraceae_UCG-001 were positively correlated with LBP, CD14, Casp12, and IL-6 gene expression and negatively correlated with CCL28, and the gut microbiota may be involved in immune regulation by influencing the inflammatory response and participate in immune regulation. In addition, the PPI network of 93 inflammation-related genes (**Figure 2J**) showed that 10 hub genes (Il6, Mmp9, Icam1, Pparg, Thbs1, Pdgfrb, Cdh1, Edn1, Ccn2, Fgf17) were at the core of the network. Combined with the differentially expressed ion channel genes (SCN8A, KCNJ13, KCNV2, RYR1, GABRB2, CHRNA6, GJB1, CACNA1D, ANO6, KCNJ14, GRIN2B, RYR2, CLIC1, CLIC2, GABRP, PKD2, KCNMA1, BEST1) from transcriptome sequencing, we performed transcription factor prediction and interactions network analysis through the JASPAR database using the NetworkAnalyst tool. The results revealed that these genes shared 39 common transcription factors (**Figure 2K**), including key factors such as NF-κB1, STAT3, and CREB1, which are closely related to the inflammatory pathway and the regulation of neural excitatory/inhibitory balance.

### 3.3 Barrier disruption and systemic inflammation driven by gut microbiota dysbiosis

To evaluate the impact of gut microbiota dysbiosis on barrier integrity and to determine the restorative potential of FMT, morphological and molecular changes in colonic and brain tissues were analyzed across CPF group and CTRL group.

**Figure 3:**
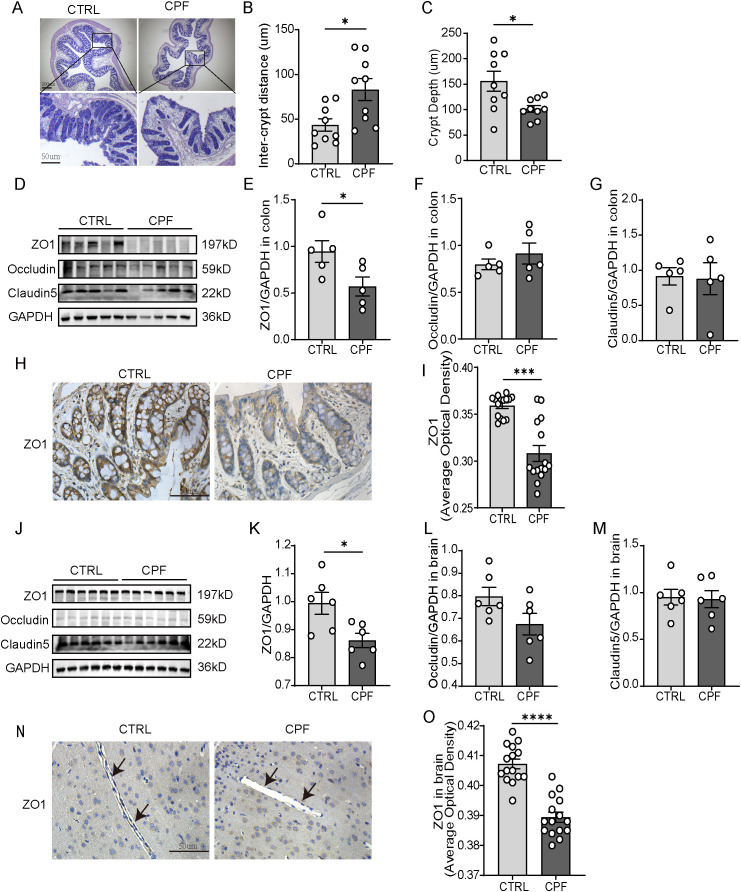
Barrier disruption and systemic inflammation driven by gut microbiota dysbiosis. **(A)** HE staining of colonic tissue. **(B, C)** Quantification of histological parameters (n = 3 per group; three random inter-crypts distance, crypts per sample): **(B)** Inter-crypts distance, **(C)** Crypt depth. **(D–G)** Western blot analysis of colonic tight junction proteins (ZO-1, Occludin, Claudin-5) (n = 5 per group). **(H, I)** Immunohistochemical staining and quantification of ZO-1 in colonic tissue (n = 3 per group; five random fields per sample). **(J–M)** Western blot analysis of tight junction proteins (ZO-1, Occludin, Claudin-5) in brain tissue (n = 6 per group). **(N, O)** Immunohistochemical staining and quantification of ZO-1 in brain tissue (n = 3 per group; five random fields per sample). Data are presented as mean ± SEM. Statistical analysis: Student’s t-test. Significance levels: *P < 0.05, **P < 0.01, ***P < 0.001, ****P < 0.0001.

Histological analysis of colonic tissue (HE staining) revealed significant morphological disruptions in CPF-treated rats (**Figure 3A**). Compared to the CTRL group, the CPF group displayed wider inter-crypts distance (P = 0.013, **Figure 3B**), reduced crypt depth (P = 0.018, **Figure 3C**). Inflammatory cell infiltration in the mucosa and submucosa and villus architectural damage were also observed in the CPF group (**Supplementary Figure 2A**). These morphological alterations indicate severe intestinal barrier damage caused by gut microbiota dysbiosis. Further analysis of tight junction proteins (TJPs) expression confirmed the loss of intestinal barrier integrity in the CPF group. Western blot (**Figures 3D–G**) and immunohistochemical staining (**Figures 3H, I**) showed significantly reduced ZO-1 levels in the CPF group compared to the CTRL group (WB P = 0.042, IHC P<0.0001). These findings demonstrate that gut microbiota dysbiosis compromises intestinal barrier function. Similarly, gut microbiota dysbiosis impaired the BBB. ZO-1 was reduced in the CPF group compared to the CTRL group (P = 0.017, **Figures 3J–M**). Immunohistochemical staining further confirmed the loss of BBB integrity, showing decreased ZO-1 expression (P<0.0001, **Figures 3N, O**). These findings imply that gut microbiota dysbiosis leads to greater BBB permeability.

**Figure 4:**
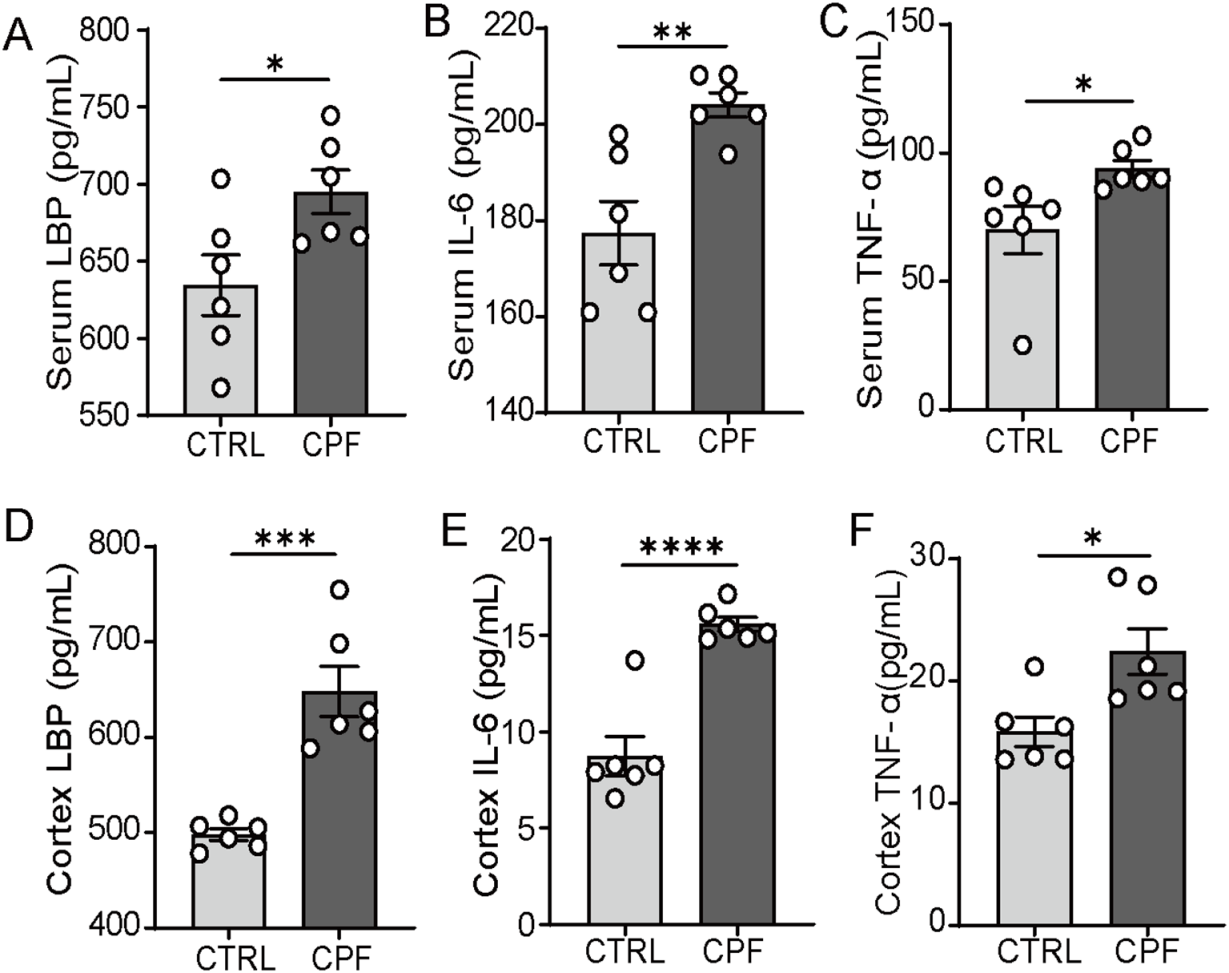
Inflammation driven by gut microbiota dysbiosis. **(A–C)** Serum levels of LBP, IL-6, and TNF-α (n = 6 per group). **(D–F)** Cortex levels of LBP, IL-6, and TNF-α (n = 6 per group). Data are presented as mean ± SEM. Statistical analysis: Student’s t-test. Significance levels: *P < 0.05, **P < 0.01, ***P < 0.001, ****P < 0.0001.

To assess systemic inflammation, we measured levels of LBP, IL-6, and TNF-α both in serum and cortex. In the CPF group, these inflammatory markers were elevated compared to the CTRL group (P = 0.031, P = 0.004, P = 0.036, respectively; **Figures 4A–C**). Compared to the CTRL group, the levels of LBP, IL-6, and TNF-α were significantly elevated in the cerebral cortex of CPF group rats (P < 0.001, P < 0.0001, and P = 0.014, respectively; **Figures 4D–F**).

### 3.4 Glial activation driven by gut microbiota dysbiosis

**Figure 5:**
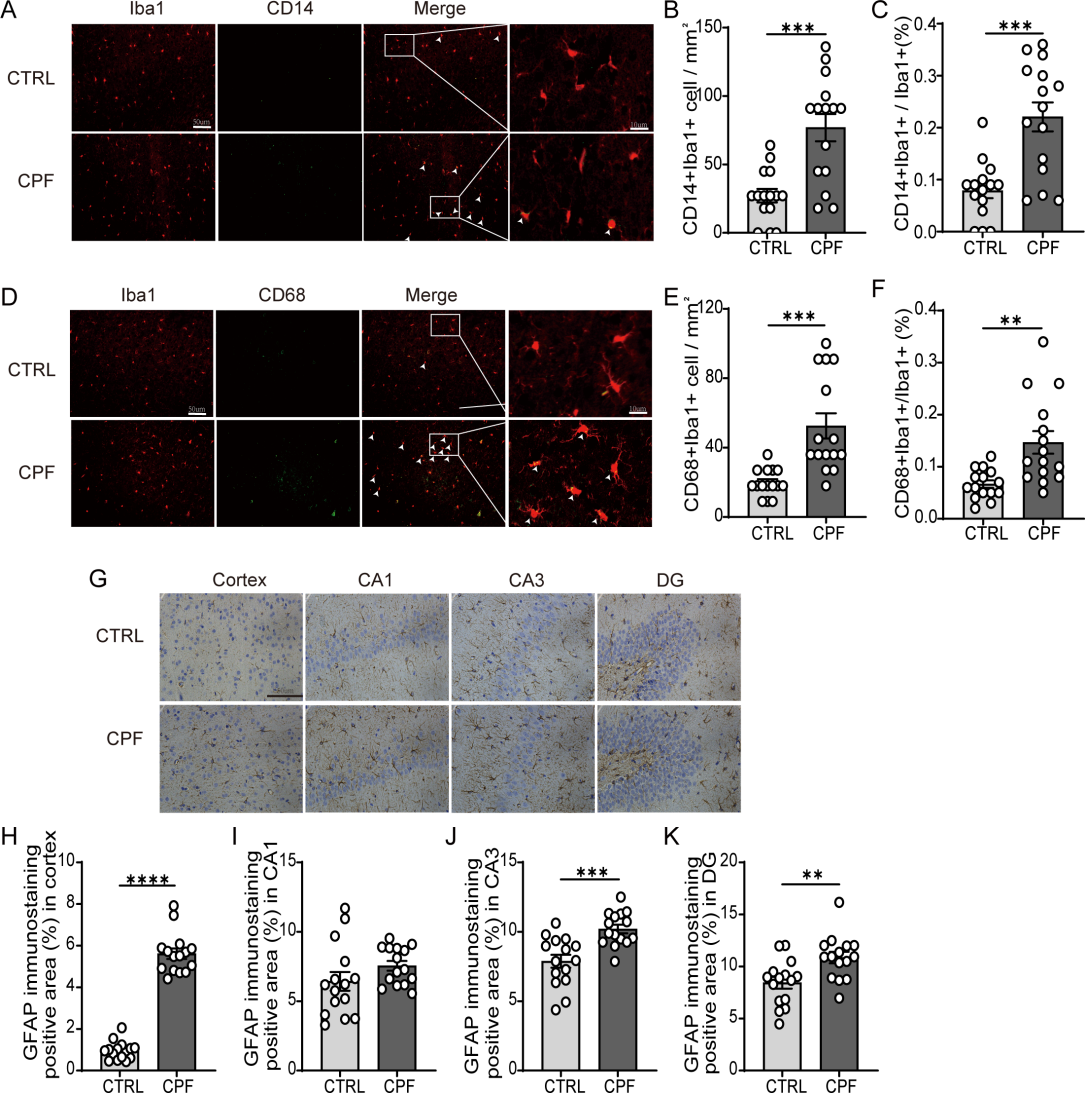
Glial activation driven by gut microbiota dysbiosis. **(A)** Immunofluorescence co-staining of Iba1 and CD14 in the cortex, with white arrows indicating Iba1^+^CD14^+^ microglia. **(B, C)** Quantification of Iba1^+^CD14^+^ microglia: number **(B)** and proportion **(C)**. **(D)** Immunofluorescence co-staining of Iba1 and CD68 in the cortex, with white arrows indicating Iba1^+^CD68^+^ microglia. **(E, F)** Quantification of Iba1^+^CD68^+^ microglia: number **(E)** and proportion **(F)**. **(G)** GFAP immunohistochemical staining in the cortex. **(H–K)** Quantification of GFAP-positive areas in the cortex, CA1, CA3, and DG regions (N = 3 per group; five random fields per sample). Data are mean ± SEM; Student’s t-test was used for Significance levels: *P < 0.05, **P < 0.01, ***P < 0.001, ****P < 0.0001.

To investigate whether gut microbiota dysbiosis affects glial cell activation in the brain, we performed immunohistochemical staining of Iba1-labeled microglia (**Supplementary Figure 3A**) and immunofluorescence co-staining of Iba1 with CD14 (**Figure 5A**), as well as Iba1 with CD68 (**Figure 5D**), in cortical tissues. Immunohistochemical staining of Iba1-labeled microglia revealed a statistically significant increase in the number of cortical microglia in the CPF group compared to the CTRL group (**Supplementary Figure 3B**). Iba1 and CD14 co-staining showed a marked increase in Iba1+CD14+ microglia in the CPF group relative to the CTRL group (P = 0.0001; **Figure 5B**). Additionally, the proportion of Iba1+CD14+/Iba1+ cells was significantly more abundant in the CPF group (P = 0.0001; **Figure 5C**). Co-staining with Iba1 and CD68, a marker of activated microglia, showed a significant increase in the number of Iba1+CD68+ microglia in the CPF group compared to the CTRL group (P < 0.001; **Figure 5E**). The proportion of Iba1+CD68+/Iba1+ cells was also elevated in the CPF group (P = 0.002; **Figure 5F**).

We also evaluated astrocytic activation using GFAP immunohistochemistry(**Figure 5G**). In the CPF group, significant astrocytic proliferation was observed in the cortex, hippocampal CA3 and hippocampal DG region compared to the CTRL group (P < 0.0001, P = 0.0003, P = 0.0048, respectively; **Figures 5H, J, K**). In the hippocampal CA1 regions, GFAP staining intensity showed no significant variation between the CPF and CTRL groups (**Figure 5I**).

### 3.5 Gut microbiota disruption and seizure susceptibility restored by FMT but not naturally

**Figure 6:**
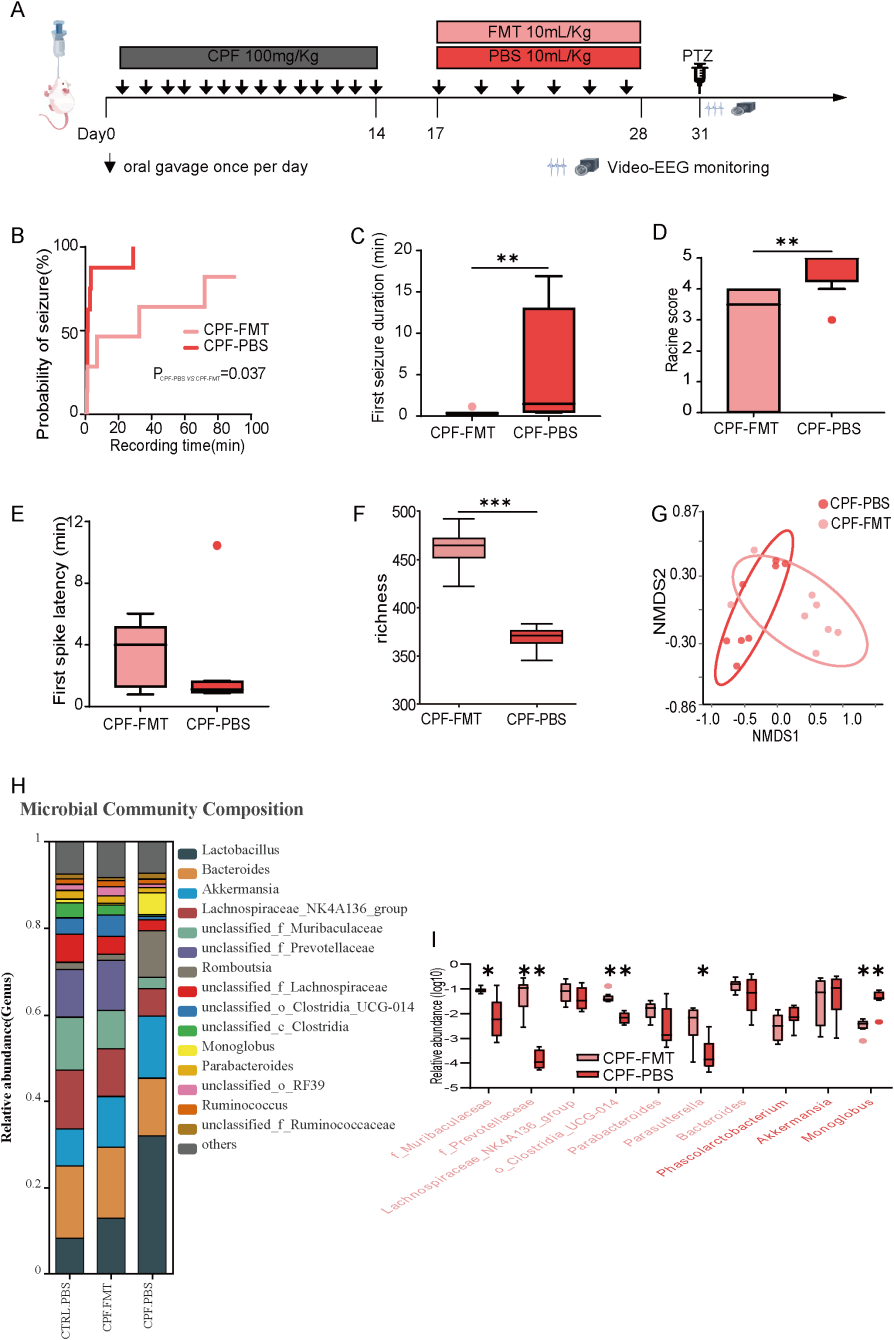
Gut Microbiota Dysbiosis and Seizure Susceptibility Restored by FMT. **(A)** Experimental timeline. **(B)** Kaplan-Meier survival curve for seizure latency. **(C)** Seizure duration of the first seizure with Racine score ≥3. **(D)** Median Racine stage. **(E)** Latency to first spike. **(F)** Alpha diversity (richness index) after 28 days of treatment. **(G)** NMDS plot showing distinct clustering between CPF-FMT and CPF-PBS groups. **(H)** Microbial community composition. **(I)** Relative abundance of the top 10 differentially abundant taxa between CPF and CTRL, compared in CPF-FMT (soft red) and CPF-PBS (deep red) groups. **(A–I)** n = 8 per group. Statistical analysis: Mann-Whitney U test. Significance levels: *P < 0.05, **P < 0.01, ***P < 0.001, ****P < 0.0001.

To validate the contribution of gut microbiota dysbiosis to increased seizure susceptibility, FMT was employed to modulate the gut microbiota in CPF group rats. An overview of the experimental design is shown in **Figure 6A**.

We compared seizure characteristics between CPF-FMT and CPF-PBS groups. Kaplan-Meier survival analysis demonstrated a significantly longer seizure latency in the CPF-FMT group (50.50 min vs. 1.28 min, Log-rank P = 0.037; **Figure 6B**). Moreover, the CPF-FMT group showed a shorter duration of first seizures (0.37 min vs. 1.48 min, P = 0.002; **Figure 6C**) and a lower median Racine stage (3.50; IQR: 0–4.00) than the CPF-PBS group (5.00; IQR: 4.25–5.00; P = 0.005; **Figure 6D**). Notably, the latency to the first spike was significantly longer in the CPF-FMT group (3.40 min vs. 1.11 min, P = 0.002; **Figure 6E**), indicating partial restoration of gut microbiota may alleviate seizure susceptibility.

On Day 28, the CPF-FMT group exhibited alpha and beta diversity fully restored to levels equivalent to those of the donor group (CTRL-PBS) (P > 0.05, **Supplementary Figure 1C, D**). The genus-level microbial composition in the CPF-FMT group closely resembled that of the CTRL-PBS group, confirming successful colonization following FMT (**Figure 6H**). The CPF-FMT group showed significantly higher alpha diversity than the CPF-PBS group (P<0.001, **Figure 6F**). NMDS analysis showed distinct clustering between CPF-FMT and CPF-PBS groups (**Figure 6G**), and significant differences in genus-level microbial composition persisted between the CPF-FMT and CPF-PBS groups, demonstrating that gut microbiota dysbiosis could not fully recover without FMT intervention (**Figure 6H** and **Supplementary Figure 1E**). Importantly, FMT reversed the depletion of f_Muribaculaceae, f_Prevotellaceae, Lachnospiraceae_NK4A136_group, o_Clostridia_UCG-014, and *Parasutterella* compared to the CPF-PBS group. Although the relative abundance of *Bacteroides* increased in the CPF-FMT group, no significant difference was identified compared to the CPF-PBS group (**Figures 6H, I**). These changes suggest partial restoration of gut microbial composition following FMT.

### 3.6 FMT alleviated barrier disruption and systemic inflammation

**Figure 7:**
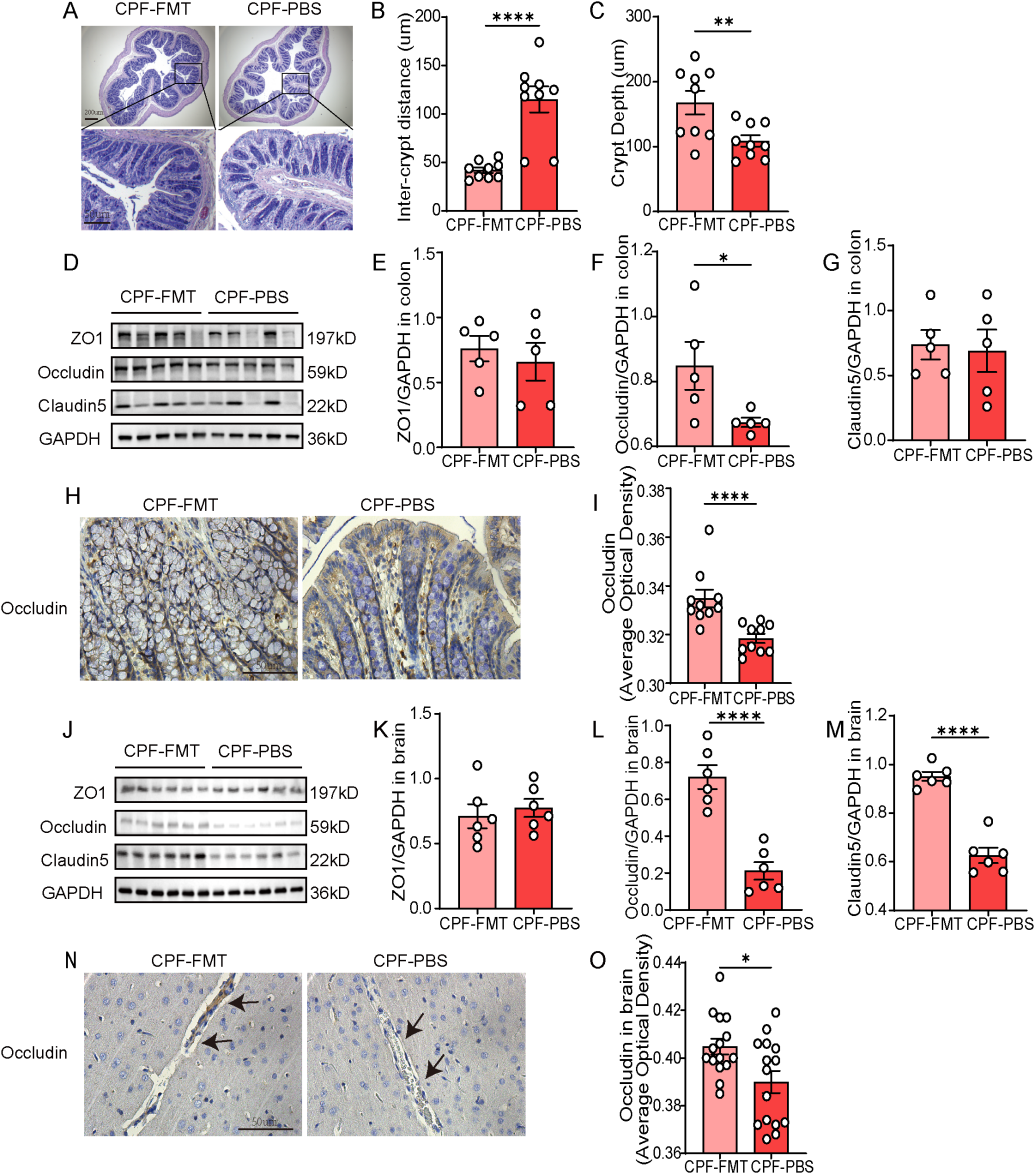
FMT alleviated barrier disruption and systemic inflammation. **(A)** HE staining of colonic tissue. **(B, C)** Quantification of histological parameters (n = 3 per group; three random inter-crypt distance, crypts per sample): **(B)** Inter-crypt distance, **(C)** Crypt depth. **(D–G)** Western blot analysis of colonic tight junction proteins (ZO-1, Occludin, Claudin-5) (n = 5 per group). **(H, I)** Immunohistochemical staining and quantification of ZO-1 in colonic tissue (n = 3 per group; five random fields per sample). **(J–M)** Western blot analysis of tight junction proteins (ZO-1, Occludin, Claudin-5) in brain tissue (n = 6 per group). **(N, O)** Immunohistochemical staining and quantification of Occludin in brain tissue (n = 3 per group; five random fields per sample). Data are presented as mean ± SEM. Statistical analysis: Student’s t-test. Significance levels: *P < 0.05, **P < 0.01, ***P < 0.001, ****P < 0.0001.

In the CPF-FMT group, inter-crypts distance and crypt depth were restored to near normal levels (P = 0.010, P<0.0001, **Figures 7A–C**). However, CPF-PBS rats exhibited persistent disruption of tight junction proteins, including reduced Occludin expression, as evidenced by Western blot and immunohistochemical analysis (P = 0.049, P<0.0001, **Figures 7D–I**). FMT effectively restored BBB function. In the CPF-FMT group, TJPs showed significantly higher expression levels relative to the CPF-PBS group (**Figures 7J–M**). Prolonged gut microbiota dysbiosis in the CPF-PBS group led to further reductions in Occludin and Claudin-5 expression (P<0.0001, P<0.0001, respectively), highlighting the necessity of gut microbiota restoration for BBB integrity. Immunohistochemical staining further confirmed the loss of BBB integrity, showing decreased Occludin expression (P = 0.014, **Figures 7N, O**).

**Figure 8:**
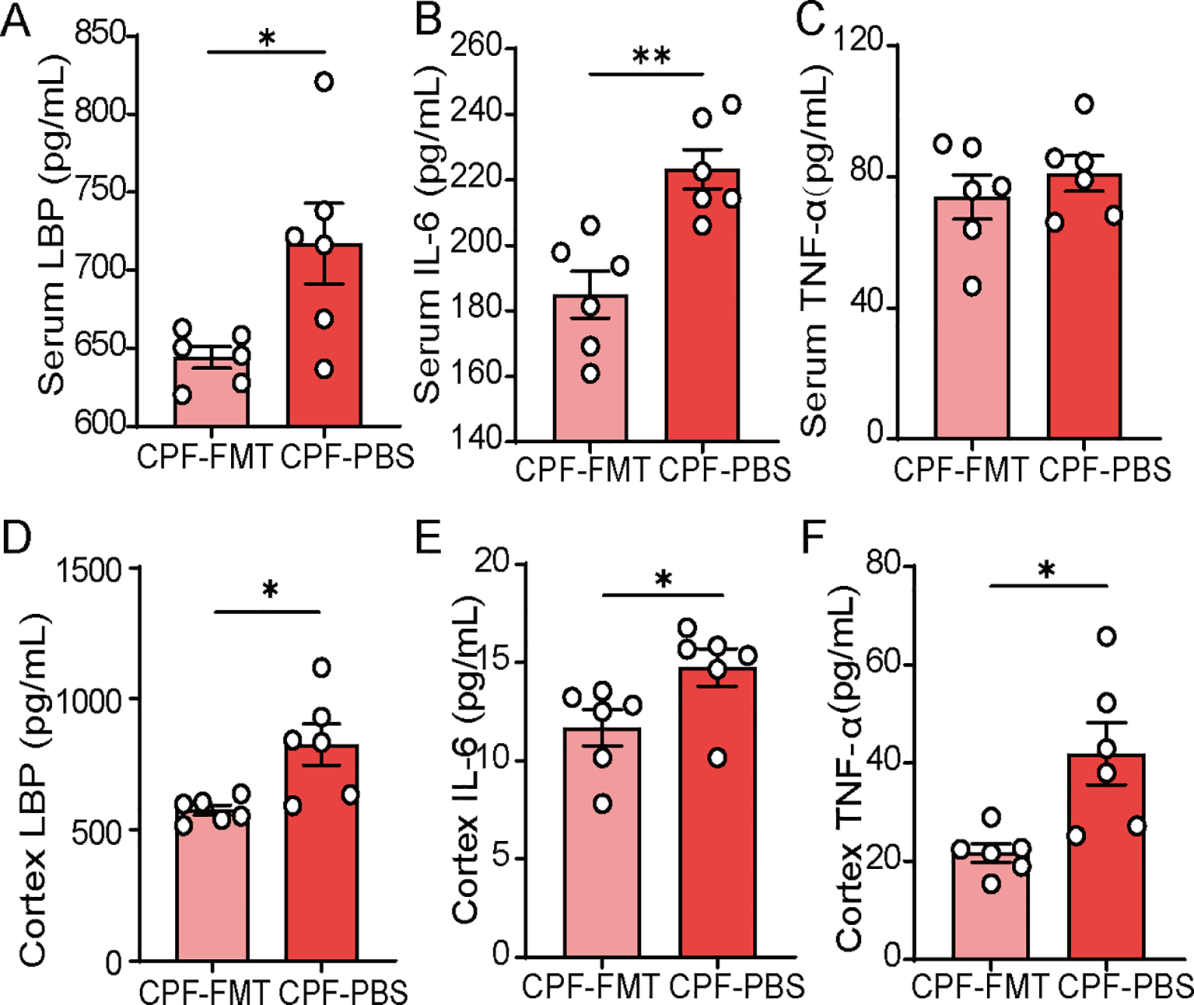
Inflammation driven by gut microbiota dysbiosis. **(A–C)** Serum levels of LBP, IL-6, and TNF-α (n = 6 per group). **(D–F)** Cortex levels of LBP, IL-6, and TNF-α (n = 6 per group). Data are presented as mean ± SEM. Statistical analysis: Student’s t-test. Significance levels: *P < 0.05, **P < 0.01, ***P < 0.001, ****P < 0.0001.

FMT reduced systemic inflammation in CPF-treated rats. Serum LBP and IL-6 levels were markedly lower in the CPF-FMT group relative to the CPF-PBS group (P = 0.021, P = 0.002, respectively; **Figures 8A, B**), while TNF-α levels showed a decreasing trend but no significant difference between CPF-FMT and CPF-PBS groups (P = 0.419, **Figure 8C**). Compared to the CPF-PBS group, the levels of LBP, IL-6, and TNF-α were significantly lower in the cerebral cortex of CPF-FMT group rats (P = 0.012, P = 0.044 and P = 0.012, respectively; **Figures 8D–F**).

### 3.7 FMT mitigates gut microbiota dysbiosis-induced glial activation

**Figure 9:**
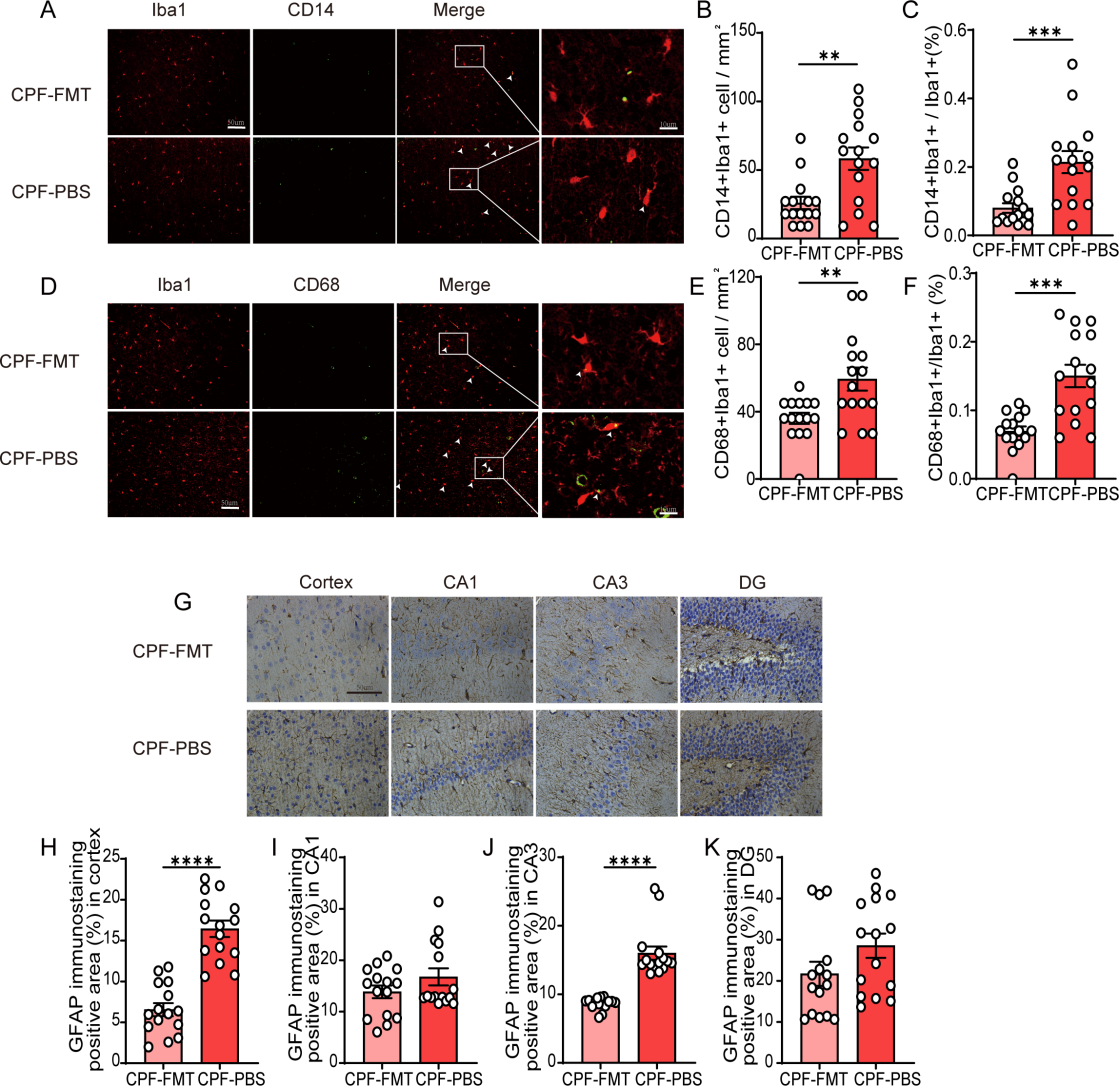
FMT mitigates gut microbiota dysbiosis-induced glial activation. **(A)** Immunofluorescence co-staining of Iba1 and CD14 in the cortex, with white arrows indicating Iba1^+^CD14^+^ microglia. **(B, C)** Quantification of Iba1^+^CD14^+^ microglia: number **(B)** and proportion **(C)**. **(D)** Immunofluorescence co-staining of Iba1 and CD68 in the cortex, with white arrows indicating Iba1^+^CD68^+^ microglia. **(E, F)** Quantification of Iba1^+^CD68^+^ microglia: number **(E)** and proportion **(F)**. **(G)** GFAP immunohistochemical staining in the cortex. **(H–K)** Quantification of GFAP-positive areas in the cortex, CA1, CA3, and DG regions (N = 3 per group; five random fields per sample). Data are mean ± SEM; Student’s t-test was used for Significance levels: *P < 0.05, **P < 0.01, ***P < 0.001, ****P < 0.0001.

Immunohistochemical staining of Iba1-labeled microglia revealed a statistically significant decrease in the number of cortical microglia in the CPF-FMT group compared to the CPF-PBS group (**Supplementary Figure 4A, B**). CPF-FMT rats exhibited reduced numbers and proportions of Iba1+CD14+ microglia compared to the CPF-PBS group (P = 0.002, P < 0.001, respectively; **Figures 9A–C**), as well as numbers and proportions of Iba1+CD68+ microglia compared to CPF-PBS rats (P = 0.005, P = 0.0001, respectively; **Figures 9D–F**). CPF-FMT rats exhibited reduced astrocytic proliferation compared to the CPF-PBS group in both cortex and hippocampal CA3 regions (P < 0.001, P < 0.0001, respectively; **Figures 9G, H, J**). While FMT reduced astrocytic activation in the CA1 and DG region compared to the CPF-PBS group, the difference was not statistically significant (P = 0.169, P = 0.112; **Figures 9I, K**).

## 4 Discussion

This study suggests that ciprofloxacin-induced gut microbiota dysbiosis compromises intestinal and blood-brain barrier integrity, promotes neuroinflammation, and increases seizure susceptibility. Importantly, FMT effectively mitigated these effects by restoring barrier function and alleviating glial activation. These findings provide new insights into the GBA and highlight the potential of microbiota-targeted interventions in modulating neuroinflammatory and neurological disorders. While previous studies have revealed a potential connection between gut microbiota and epilepsy, particularly through the GBA, the specific mechanisms underlying this association remain incompletely understood (8, 11, 31–36). Most existing research has primarily focused on observational or correlational evidence, with limited experimental validation or mechanistic insights (32, 34–37). Our study addresses this gap by providing evidence that gut microbiota dysbiosis triggers neuroinflammation and barrier dysfunction, contributing to seizure susceptibility. This study highlights the potential relevance of microbiota-targeted interventions in experimental epilepsy models, contributing to our understanding of the GBA in seizure, while also addressing the impact of antibiotic-induced microbiota alterations on health.

Various experimental epilepsy models have been widely used to investigate seizure mechanisms, including PTZ kindling, kainic acid (KA), pilocarpine-induced status epilepticus, and genetic models (38). These models primarily focus on neuronal hyperexcitability and network remodeling. In contrast, our ciprofloxacin-induced dysbiosis model emphasizes the contribution of microbiota perturbation as a modifiable environmental factor influencing seizure susceptibility.

Our findings reveal that gut microbiota dysbiosis significantly enhances seizure susceptibility, as evidenced by shorter seizure latency, higher Racine stage, and reduced spike-wave discharge latency in dysbiotic rats. Although ciprofloxacin is known to exert direct neurotoxic and GABA-A antagonistic effects, previous work from our group using an identical experimental paradigm did not detect significant differences in ciprofloxacin concentrations in brain tissue between treated and control rats (15). Ciprofloxacin levels were not measured in the present cohort, which represents a limitation of this study. The association between FMT and attenuation of these effects by FMT supports an important association of gut microbiota in modulating seizure susceptibility (9, 37, 39, 40). Gut microbiota dysbiosis-induced disruptions in systemic homeostasis, including compromised intestinal and blood-brain barrier integrity and heightened neuroinflammation, have been implicated in epilepsy pathogenesis (41, 42). The inability of the gut microbiota to recover naturally in the CPF-PBS group further highlights the persistent impact of gut microbiota dysbiosis on neurophysiological processes. This finding aligns with previous studies demonstrating that antibiotic-induced gut microbiota dysbiosis often leads to prolonged disruptions in gut microbiota composition, with limited potential for natural recovery (43–46). Furthermore, the persistent nature of gut microbiota dysbiosis and its systemic effects on host physiology have been extensively reported (47, 48), and our study further elucidates its specific impact on seizure susceptibility. By showing that microbiota restoration is associated with reduced seizure susceptibility, our study supports a relationship between gut microbiota dysbiosis and seizure, emphasizing the therapeutic potential of microbiota-targeted interventions in epilepsy.

In our study, gut microbiota dysbiosis was characterized by a significant reduction in microbial diversity and the depletion of putatively beneficial taxa, such as f_Muribaculaceae, f_Prevotellaceae, *Parasutterella*, and Lachnospiraceae_NK4A136_group, which are integral to gut barrier function, short-chain fatty acid (SCFA) production, and immune modulation (49–55). *Akkermansia* has been shown to exert beneficial effects on epilepsy in some studies (9), while others have associated it with epileptic disease states (56–59). These apparently contradictory findings suggest that the role of Akkermansia in epilepsy is context-dependent (60) and may reflect a compensatory response to antibiotic-induced barrier disruption and inflammation under dysbiotic conditions, rather than a uniform protective or pathogenic effect. The observed increase in *Akkermansia* in the CPF group may reflect a complex interplay between gut microbiota dysbiosis and host response, warranting further investigation into its role in seizure susceptibility. Notably, *Parabacteroides* was significantly depleted in dysbiotic rats. Previous studies have highlighted the role of *Parabacteroides* in mediating anti-seizure effects through the GBA, as evidenced by its neuroprotective properties in epilepsy models (9). Additionally, its role in modulating immune responses and maintaining gut homeostasis may further contribute to neurological health, including potential neuroprotective effects (6, 41). Its depletion in our CPF-treated group may contribute to compromised gut barrier integrity and subsequent inflammatory activation.

A key feature of gut microbiota dysbiosis is the overgrowth of Gram-negative bacteria that produce LPS (61, 62). LPS is a major component of the outer membrane of Gram-negative bacteria, which exhibits strong pro-inflammatory properties and exerts deleterious effects on gut barrier function by disrupting TJPs and promoting intestinal inflammation (62). Although LPS is a key mediator of immune responses induced by gut microbiota dysbiosis, its blood concentration is typically low and susceptible to sampling timing and technical factors, leading to poor reproducibility. Therefore, LBP was used as an indirect indicator of LPS exposure. Previous studies have shown that LBP levels closely correlate with LPS-induced immune load and more stably reflect systemic inflammation (30). Through binding to LBP and co-receptor CD14, LPS activates downstream inflammatory signaling cascades (63, 64). These pathways have been associated with systemic inflammation, BBB destruction, and neuroinflammation (30, 65, 66). In our study, rats with gut microbiota dysbiosis exhibited elevated levels of inflammatory cytokines in both serum and brain tissues, along with a reduction in the expression of tight junction proteins in the intestinal and BBB. Inflammation has been recognized as a key driver of BBB disruption (67). Specifically, LPS binding to LBP and CD14 triggers an inflammatory cascade that ultimately compromises BBB integrity. Notably, Lbp−/− and Cd14−/− mice display resistance to BBB disruption (65). Peripheral inflammation has been shown to modulate BBB permeability, promoting microglial and astrocytic activation, which in turn exacerbates neuroinflammation and perpetuates BBB dysfunction (68). Thus, the neuroinflammation observed in our study may act as both a consequence and a driving force of BBB impairment, establishing a feedforward loop that exacerbates CNS pathology.

In our study, astrocyte proliferation in rats with gut microbiota dysbiosis was not restricted to the cortical regions but was also significantly increased in the hippocampal CA3 and dentate gyrus (DG) regions. In contrast, microglial expansion was predominantly observed in the cortex. The activation of microglia and astrocytes has been implicated in seizure generation (68). Previous studies have shown that excessive astrocyte activation can exacerbate neural dysfunction by amplifying local immune responses and interacting synergistically with microglia to intensify neuroinflammation (69). In the present study, the activation of these glial cells may represent a key factor contributing to the increased seizure susceptibility observed in the gut microbiota dysbiosis model. It has been established that microglia exhibit distinct transcriptional profiles and functional regulatory mechanisms across different brain regions (70). In contrast, astrocyte proliferation is regulated not only by inflammatory signals but also by synaptic plasticity and neuronal excitability (71). Thus, the expansion of astrocytes in the hippocampus may be closely linked to excitotoxicity and synaptic homeostasis rather than being a mere passive response to inflammatory signaling. The region-specific activation patterns of microglia and astrocytes in response to gut microbiota dysbiosis warrant further investigation as a potential mechanistic link between neuroinflammation and seizure susceptibility.

Importantly, FMT was associated with improvement in these pathological changes, restoring microbial diversity and the abundance of key protective taxa. This restoration coincided with improved tight junction protein expression in both the intestinal and BBB contexts, highlighting the critical role of a balanced microbiota in maintaining barrier integrity (5, 72–74). In contrast, the CPF-PBS group exhibited persistent barrier dysfunction and systemic inflammation, demonstrating the resilience of gut microbiota dysbiosis and its inability to recover naturally (75, 76). Given the broad nature of FMT, causal contributions of individual microbial taxa or metabolites cannot be determined in the present study. This limitation is consistent with previous reviews emphasizing that FMT represents a global microbiota intervention and that disentangling causality of specific taxa in epilepsy remains challenging (60).

Despite these significant findings, our study has limitations. Transcriptomic and histological analyses were conducted with small sample sizes as exploratory, hypothesis-generating experiments, which may limit statistical power. Therefore, these findings should be interpreted with caution and require validation in larger cohorts. While our results establish a strong association between gut microbiota dysbiosis and neuroinflammation, the causal mechanisms linking specific microbial taxa to glial activation require further investigation. Future studies couldn’t explore targeted microbiota interventions, such as the use of specific probiotics or microbial metabolites, to better understand their therapeutic potential in neurological disorders.

## 5 Conclusion

This study provides evidence suggesting that gut microbiota is related to seizure susceptibility through neuroinflammation in the GBA. By restoring gut microbiota composition, particularly through FMT, this study highlights the potential relevance of microbiota-targeted interventions in modulating neuroinflammation and barrier dysfunction. These findings advance our understanding of the GBA in fluoroquinolone-induced seizures. However, this study is based on a single antibiotic–induced gut microbiota dysbiosis model in rodents with limited sample sizes, and its findings should not be directly extrapolated to human epilepsy. Future research should focus on clinical validation of these findings and explore the broader implications of microbiota modulation in neuroinflammatory diseases.

## Data Availability

The RNA-seq data generated in this study have been deposited in the NCBI Sequence Read Archive (SRA) under BioProject (https://dataview.ncbi.nlm.nih.gov/object/PRJNA1438453?reviewer=if7bh4b6cudj8ah7jpbpju09rc) accession number PRJNA1438453.

## References

[1] Asadi-Pooya AA Brigo F Lattanzi S Blumcke I . Adult epilepsy. Lancet. (2023) 402:412–24. doi: 10.1016/S0140-6736(23)01048-6, PMID: 37459868

[2] Fisher RS Acevedo C Arzimanoglou A Bogacz A Cross JH Elger CE . ILAE official report: a practical clinical definition of epilepsy. Epilepsia. (2014) 55:475–82. doi: 10.1111/epi.12550, PMID: 24730690

[3] Agirman G Yu KB Hsiao EY . Signaling inflammation across the gut-brain axis. Science. (2021) 374:1087–92. doi: 10.1126/science.abi6087, PMID: 34822299

[4] Agirman G Hsiao EY . SnapShot: The microbiota-gut-brain axis. Cell. (2021) 184:2524. doi: 10.1016/j.cell.2021.03.022, PMID: 33930299

[5] Cryan JF O'Riordan KJ Cowan CSM Sandhu KV Bastiaanssen TFS Boehme M . The microbiota-gut-brain axis. Physiol Rev. (2019) 99:1877–2013. doi: 10.1152/physrev.00018.2018, PMID: 31460832

[6] Fung TC Olson CA Hsiao EY . Interactions between the microbiota, immune and nervous systems in health and disease. Nat Neurosci. (2017) 20:145–55. doi: 10.1038/nn.4476, PMID: 28092661 PMC6960010

[7] Dalile B Van Oudenhove L Vervliet B Verbeke K . The role of short-chain fatty acids in microbiota-gut-brain communication. Nat Rev Gastroenterol Hepatol. (2019) 16:461–78. doi: 10.1038/s41575-019-0157-3, PMID: 31123355

[8] Sorboni SG Moghaddam HS Jafarzadeh-Esfehani R Soleimanpour S . A comprehensive review on the role of the gut microbiome in human neurological disorders. Clin Microbiol Rev. (2022) 35:e33820. doi: 10.1128/CMR.00338-20, PMID: 34985325 PMC8729913

[9] Olson CA Vuong HE Yano JM Liang QY Nusbaum DJ Hsiao EY . The gut microbiota mediates the anti-seizure effects of the ketogenic diet. Cell. (2018) 173:1728–41. doi: 10.1016/j.cell.2018.04.027, PMID: 29804833 PMC6003870

[10] Mejía-Granados DM Villasana-Salazar B Lozano-García L Cavalheiro EA Striano P . Gut-microbiota-directed strategies to treat epilepsy: clinical and experimental evidence. Seizure. (2021) 90:80–92. doi: 10.1016/j.seizure.2021.03.009, PMID: 33762166

[11] Ding M Lang Y Shu H Shao J Cui L . Microbiota-gut-brain axis and epilepsy: A review on mechanisms and potential therapeutics. Front Immunol. (2021) 12:742449. doi: 10.3389/fimmu.2021.742449, PMID: 34707612 PMC8542678

[12] Tiwari P Dwivedi R Bansal M Tripathi M Dada R . Role of gut microbiota in neurological disorders and its therapeutic significance. J Clin Med. (2023) 12:1650. doi: 10.3390/jcm12041650, PMID: 36836185 PMC9965848

[13] Riva A Pozzati E Grasso M De Caro C Russo E Verrotti A . Targeting the MGBA with -biotics in epilepsy: New insights from preclinical and clinical studies. Neurobiol Dis. (2022) 170:105758. doi: 10.1016/j.nbd.2022.105758, PMID: 35588991

[14] Lum GR Olson CA Hsiao EY . Emerging roles for the intestinal microbiome in epilepsy. Neurobiol Dis. (2020) 135:104576. doi: 10.1016/j.nbd.2019.104576, PMID: 31445165

[15] Zou S Li Y Zou Q Yang M Li H Niu R . Gut microbiota and serum metabolomic alterations in modulating the impact of fecal microbiota transplantation on ciprofloxacin-induced seizure susceptibility. Front Microbiol. (2024) 15:1403892. doi: 10.3389/fmicb.2024.1403892, PMID: 38962126 PMC11220169

[16] Xie G Zhou Q Qiu CZ Dai WK Wang HP Li YH . Ketogenic diet poses a significant effect on imbalanced gut microbiota in infants with refractory epilepsy. World J Gastroenterol. (2017) 23:6164–71. doi: 10.3748/wjg.v23.i33.6164, PMID: 28970732 PMC5597508

[17] Peng A Qiu X Lai W Li W Zhang L Zhu X . Altered composition of the gut microbiome in patients with drug-resistant epilepsy. Epilepsy Res. (2018) 147:102–7. doi: 10.1016/j.eplepsyres.2018.09.013, PMID: 30291996

[18] Şafak B Altunan B Topçu B Eren Topkaya A . The gut microbiome in epilepsy. Microb Pathog. (2020) 139:103853. doi: 10.1016/j.micpath.2019.103853, PMID: 31730997

[19] Holmes M Flaminio Z Vardhan M Xu F Li X Devinsky O . Cross talk between drug-resistant epilepsy and the gut microbiome. Epilepsia. (2020) 61:2619–28. doi: 10.1111/epi.16744, PMID: 33140419

[20] Oliveira MET Paulino GVB Dos Santos Júnior ED da Silva Oliveira FA Melo VMM Ursulino JS . Multi-omic analysis of the gut microbiome in rats with lithium-pilocarpine-induced temporal lobe epilepsy. Mol Neurobiol. (2022) 59:6429–46. doi: 10.1007/s12035-022-02984-3, PMID: 35962889

[21] Eor JY Tan PL Son YJ Kwak MJ Kim SH . Gut microbiota modulation by both Lactobacillus fermentum MSK 408 and ketogenic diet in a murine model of pentylenetetrazole-induced acute seizure. Epilepsy Res. (2021) 169:106506. doi: 10.1016/j.eplepsyres.2020.106506, PMID: 33276243

[22] Tahmasebi S Oryan S Mohajerani HR Akbari N Palizvan MR . Probiotics and Nigella sativa extract supplementation improved behavioral and electrophysiological effects of PTZ-induced chemical kindling in rats. Epilepsy Behav. (2020) 104:106897. doi: 10.1016/j.yebeh.2019.106897, PMID: 32028126

[23] Bagheri S Heydari A Alinaghipour A Salami M . Effect of probiotic supplementation on seizure activity and cognitive performance in PTZ-induced chemical kindling. Epilepsy Behav. (2019) 95:43–50. doi: 10.1016/j.yebeh.2019.03.038, PMID: 31026781

[24] Gómez-Eguílaz M Ramón-Trapero JL Pérez-Martínez L Blanco JR . The beneficial effect of probiotics as a supplementary treatment in drug-resistant epilepsy: a pilot study. Benef Microbes. (2018) 9:875–81. doi: 10.3920/BM2018.0018, PMID: 30198325

[25] Kebede V Ravizza T Balosso S Di Sapia R Canali L Soldi S . Early treatment with rifaximin during epileptogenesis reverses gut alterations and reduces seizure duration in a mouse model of acquired epilepsy. Brain Behav Immun. (2024) 119:363–80. doi: 10.1016/j.bbi.2024.04.007, PMID: 38608741

[26] Kong W Mao W Zhang L Wu Y . Disproportionality analysis of quinolone safety in children using data from the FDA adverse event reporting system (FAERS). Front Pediatr. (2022) 10:1069504. doi: 10.3389/fped.2022.1069504, PMID: 36714649 PMC9874243

[27] Gueta I Yonath H Fluss R Oberman B Oppenheim A Ozeri D . Fluoroquinolones and the risk for incidental seizures: a comparative retrospective study. J Antimicrob Chemother. (2024) 79:2554–60. doi: 10.1093/jac/dkae255, PMID: 39090969

[28] Bokoliya SC Dorsett Y Panier H Zhou Y . Procedures for fecal microbiota transplantation in murine microbiome studies. Front Cell Infect Microbiol. (2021) 11:711055. doi: 10.3389/fcimb.2021.711055, PMID: 34621688 PMC8490673

[29] Gheorghe CE Ritz NL Martin JA Wardill HR Cryan JF Clarke G . Investigating causality with fecal microbiota transplantation in rodents: applications, recommendations and pitfalls. Gut Microbes. (2021) 13:1941711. doi: 10.1080/19490976.2021.1941711, PMID: 34328058 PMC8331043

[30] Zhao Y Walker DI Lill CM Bloem BR Darweesh SKL Pinto-Pacheco B . Lipopolysaccharide-binding protein and future Parkinson's disease risk: a European prospective cohort. J Neuroinflamm. (2023) 20:170. doi: 10.1186/s12974-023-02846-2, PMID: 37480114 PMC10362572

[31] Dahlin M Wheelock CE Prast-Nielsen S . Association between seizure reduction during ketogenic diet treatment of epilepsy and changes in circulatory metabolites and gut microbiota composition. EBioMedicine. (2024) 109:105400. doi: 10.1016/j.ebiom.2024.105400, PMID: 39500011 PMC11570732

[32] Lindefeldt M Eng A Darban H Bjerkner A Zetterström CK Allander T . The ketogenic diet influences taxonomic and functional composition of the gut microbiota in children with severe epilepsy. NPJ Biofilms Microbiomes. (2019) 5:5. doi: 10.1038/s41522-018-0073-2, PMID: 30701077 PMC6344533

[33] Citraro R Lembo F De Caro C Tallarico M Coretti L Iannone LF . First evidence of altered microbiota and intestinal damage and their link to absence epilepsy in a genetic animal model, the WAG/Rij rat. Epilepsia. (2021) 62:529–41. doi: 10.1111/epi.16813, PMID: 33428780

[34] Zeng Y Cao S Yang H . Roles of gut microbiome in epilepsy risk: A Mendelian randomization study. Front Microbiol. (2023) 14:1115014. doi: 10.3389/fmicb.2023.1115014, PMID: 36922970 PMC10010438

[35] Fusco F Perottoni S Giordano C Riva A Iannone LF De Caro C . The microbiota-gut-brain axis and epilepsy from a multidisciplinary perspective: Clinical evidence and technological solutions for improvement of *in vitro* preclinical models. Bioeng Transl Med. (2022) 7:e10296. doi: 10.1002/btm2.10296, PMID: 35600638 PMC9115712

[36] Ceccarani C Viganò I Ottaviano E Redaelli MG Severgnini M Vignoli A . Is gut microbiota a key player in epilepsy onset? A longitudinal study in drug-naive children. Front Cell Infect Microbiol. (2021) 11:749509. doi: 10.3389/fcimb.2021.749509, PMID: 34926315 PMC8677705

[37] He Z Cui BT Zhang T Li P Long CY Ji GZ . Fecal microbiota transplantation cured epilepsy in a case with Crohn's disease: The first report. World J Gastroenterol. (2017) 23:3565–8. doi: 10.3748/wjg.v23.i19.3565, PMID: 28596693 PMC5442093

[38] Löscher W . Animal models of seizures and epilepsy: past, present, and future role for the discovery of antiseizure drugs. Neurochem Res. (2017) 42:1873–88. doi: 10.1007/s11064-017-2222-z, PMID: 28290134

[39] Prast-Nielsen S . Fecal transfers from children on the ketogenic diet mimic the anti-seizure effect in mice. Cell Rep. (2023) 42:113570. doi: 10.1016/j.celrep.2023.113570, PMID: 38070136

[40] Li X Li J Ji J Li S Yao X Fan H . Gut microbiota modification by diosgenin mediates antiepileptic effects in a mouse model of epilepsy. J Neurochem. (2023) 168:3982–4000. doi: 10.1111/jnc.16033, PMID: 38115597

[41] Sharon G Sampson TR Geschwind DH Mazmanian SK . The central nervous system and the gut microbiome. Cell. (2016) 167:915–32. doi: 10.1016/j.cell.2016.10.027, PMID: 27814521 PMC5127403

[42] Li Q Gu Y Liang J Yang Z Qin J . A long journey to treat epilepsy with the gut microbiota. Front Cell Neurosci. (2024) 18:1386205. doi: 10.3389/fncel.2024.1386205, PMID: 38988662 PMC11233807

[43] Suez J Zmora N Zilberman-Schapira G Mor U Dori-Bachash M Bashiardes S . Post-antibiotic gut mucosal microbiome reconstitution is impaired by probiotics and improved by autologous FMT. Cell. (2018) 174:1406–23. doi: 10.1016/j.cell.2018.08.047, PMID: 30193113

[44] Wei J Liu C Qin D Ren F Duan J Chen T . Targeting inflammation and gut microbiota with antibacterial therapy: Implications for central nervous system health. Ageing Res Rev. (2024) 102:102544. doi: 10.1016/j.arr.2024.102544, PMID: 39419400

[45] Palleja A Mikkelsen KH Forslund SK Kashani A Allin KH Nielsen T . Recovery of gut microbiota of healthy adults following antibiotic exposure. Nat Microbiol. (2018) 3:1255–65. doi: 10.1038/s41564-018-0257-9, PMID: 30349083

[46] Ng KM Aranda-Díaz A Tropini C Frankel MR Van Treuren W O'Loughlin CT . Recovery of the gut microbiota after antibiotics depends on host diet, community context, and environmental reservoirs. Cell Host Microbe. (2019) 26:650–65. doi: 10.1016/j.chom.2019.10.011, PMID: 31726029 PMC8276089

[47] Anthony WE Wang B Sukhum KV D'Souza AW Hink T Cass C . Acute and persistent effects of commonly used antibiotics on the gut microbiome and resistome in healthy adults. Cell Rep. (2022) 39:110649. doi: 10.1016/j.celrep.2022.110649, PMID: 35417701 PMC9066705

[48] Korpela K Salonen A Virta LJ Kekkonen RA Forslund K Bork P . Intestinal microbiome is related to lifetime antibiotic use in Finnish pre-school children. Nat Commun. (2016) 7:10410. doi: 10.1038/ncomms10410, PMID: 26811868 PMC4737757

[49] Lagkouvardos I Lesker TR Hitch TCA Gálvez EJC Smit N Neuhaus K . Sequence and cultivation study of Muribaculaceae reveals novel species, host preference, and functional potential of this yet undescribed family. Microbiome. (2019) 7:28. doi: 10.1186/s40168-019-0637-2, PMID: 30782206 PMC6381624

[50] Lin TL Kuo YL Lai JH Lu CC Yuan CT Hsu CY . Gut microbiota dysbiosis-related susceptibility to nontuberculous mycobacterial lung disease. Gut Microbes. (2024) 16:2361490. doi: 10.1080/19490976.2024.2361490, PMID: 38860456 PMC11174134

[51] Larsen JM . The immune response to Prevotella bacteria in chronic inflammatory disease. Immunology. (2017) 151:363–74. doi: 10.1111/imm.12760, PMID: 28542929 PMC5506432

[52] De Filippis F Pellegrini N Vannini L Jeffery IB La Storia A Laghi L . High-level adherence to a Mediterranean diet beneficially impacts the gut microbiota and associated metabolome. Gut. (2016) 65:1812–21. doi: 10.1136/gutjnl-2015-309957, PMID: 26416813

[53] Chen J Mei MS Yu Y Zhao Y Gong H Chen W . Elegant approach to intervention of homogalacturonan from the fruits of Ficus pumila L. in colitis: Unraveling the role of methyl esters and acetyl groups. Int J Biol Macromol. (2024) 283:137793. doi: 10.1016/j.ijbiomac.2024.137793, PMID: 39557266

[54] Zhao Z Zhong L Zhou P Deng Y Liu G Li P . Impact of dietary fatty acid composition on the intestinal microbiota and fecal metabolism of rats fed a high-fructose/high-fat diet. Nutrients. (2024) 16:3774. doi: 10.3390/nu16213774, PMID: 39519607 PMC11547413

[55] Xi YY Chen C Zheng JJ Jiang B Dong XY Lou SY . Ampelopsis grossedentata tea alleviating liver fibrosis in BDL-induced mice via gut microbiota and metabolite modulation. NPJ Sci Food. (2024) 8:93. doi: 10.1038/s41538-024-00334-2, PMID: 39537664 PMC11561287

[56] Xu L Chen D Zhao C Jiang L Mao S Song C . Decreased abundance of Akkermansia after adrenocorticotropic hormone therapy in patients with West syndrome. BMC Microbiol. (2021) 21:126. doi: 10.1186/s12866-021-02189-z, PMID: 33892634 PMC8063292

[57] Huang C Chu C Peng Y Zhang N Yang Z You J . Correlations between gastrointestinal and oral microbiota in children with cerebral palsy and epilepsy. Front Pediatr. (2022) 10:988601. doi: 10.3389/fped.2022.988601, PMID: 36440329 PMC9686843

[58] Arulsamy A Tan QY Balasubramaniam V O'Brien TJ Shaikh MF . Gut microbiota and epilepsy: A systematic review on their relationship and possible therapeutics. ACS Chem Neurosci. (2020) 11:3488–98. doi: 10.1021/acschemneuro.0c00431, PMID: 33064448

[59] He X Zhang Y . Changes in gut flora in patients with epilepsy: a systematic review and meta-analysis. Front Microbiol. (2024) 15:1480022. doi: 10.3389/fmicb.2024.1480022, PMID: 39611090 PMC11602489

[60] Iannone LF Gómez-Eguílaz M De Caro C . Gut microbiota manipulation as an epilepsy treatment. Neurobiol Dis. (2022) 174:105897. doi: 10.1016/j.nbd.2022.105897, PMID: 36257595

[61] Weiss GA Hennet T . Mechanisms and consequences of intestinal dysbiosis. Cell Mol Life Sci. (2017) 74:2959–77. doi: 10.1007/s00018-017-2509-x, PMID: 28352996 PMC11107543

[62] Cao S Zhang Q Wang C Wu H Jiao L Hong Q . LPS challenge increased intestinal permeability, disrupted mitochondrial function and triggered mitophagy of piglets. Innate Immun. (2018) 24:221–30. doi: 10.1177/1753425918769372, PMID: 29642727 PMC6830921

[63] Yin R Wang T Dai H Han J Sun J Liu N . Immunogenic molecules associated with gut bacterial cell walls: chemical structures, immune-modulating functions, and mechanisms. Protein Cell. (2023) 14:776–85. doi: 10.1093/procel/pwad016, PMID: 37013853 PMC10599643

[64] Sharygin D Koniaris LG Wells C Zimmers TA Hamidi T . Role of CD14 in human disease. Immunology. (2023) 169:260–70. doi: 10.1111/imm.13634, PMID: 36840585 PMC10591340

[65] Wei C Jiang W Wang R Zhong H He H Gao X . Brain endothelial GSDMD activation mediates inflammatory BBB breakdown. Nature. (2024) 629:893–900. doi: 10.1038/s41586-024-07314-2, PMID: 38632402

[66] Khedr LH Eladawy RM Nassar NN Saad MAE . Canagliflozin attenuates chronic unpredictable mild stress induced neuroinflammation via modulating AMPK/mTOR autophagic signaling. Neuropharmacology. (2023) 223:109293. doi: 10.1016/j.neuropharm.2022.109293, PMID: 36272443

[67] Librizzi L Noè F Vezzani A de Curtis M Ravizza T . Seizure-induced brain-borne inflammation sustains seizure recurrence and blood-brain barrier damage. Ann Neurol. (2012) 72:82–90. doi: 10.1002/ana.23567, PMID: 22829270

[68] Onat F Andersson M Çaçak N . The role of glial cells in the pathophysiology of epilepsy. Cells. (2025) 14:94. doi: 10.3390/cells14020094, PMID: 39851521 PMC11763453

[69] Lawrence JM Schardien K Wigdahl B Nonnemacher MR . Roles of neuropathology-associated reactive astrocytes: a systematic review. Acta Neuropathol Commun. (2023) 11:42. doi: 10.1186/s40478-023-01526-9, PMID: 36915214 PMC10009953

[70] Tan YL Yuan Y Tian L . Microglial regional heterogeneity and its role in the brain. Mol Psychiatry. (2020) 25:351–67. doi: 10.1038/s41380-019-0609-8, PMID: 31772305 PMC6974435

[71] Xin W Bonci A . Functional astrocyte heterogeneity and implications for their role in shaping neurotransmission. Front Cell Neurosci. (2018) 12:141. doi: 10.3389/fncel.2018.00141, PMID: 29896091 PMC5987431

[72] Braniste V Al-Asmakh M Kowal C Anuar F Abbaspour A Tóth M . The gut microbiota influences blood-brain barrier permeability in mice. Sci Transl Med. (2014) 6:158r–263r. doi: 10.1126/scitranslmed.3009759, PMID: 25411471 PMC4396848

[73] Zhuang M Zhang X Cai J . Microbiota-gut-brain axis: interplay between microbiota, barrier function and lymphatic system. Gut Microbes. (2024) 16:2387800. doi: 10.1080/19490976.2024.2387800, PMID: 39182226 PMC11346530

[74] Rutsch A Kantsjö JB Ronchi F . The gut-brain axis: how microbiota and host inflammasome influence brain physiology and pathology. Front Immunol. (2020) 11:604179. doi: 10.3389/fimmu.2020.604179, PMID: 33362788 PMC7758428

[75] Becattini S Taur Y Pamer EG . Antibiotic-induced changes in the intestinal microbiota and disease. Trends Mol Med. (2016) 22:458–78. doi: 10.1016/j.molmed.2016.04.003, PMID: 27178527 PMC4885777

[76] Okumura R Takeda K . Maintenance of intestinal homeostasis by mucosal barriers. Inflammation Regener. (2018) 38:5. doi: 10.1186/s41232-018-0063-z, PMID: 29619131 PMC5879757

