# Supplementary material for: Ciprofloxacin-induced microbiota dysbiosis triggers seizure susceptibility through the microbiota-gut-brain axis
Source: Front Immunol. 2026 Mar 31;17:1670694. doi: 10.3389/fimmu.2026.1670694 (PMC13076159; doi:10.3389/fimmu.2026.1670694)
Supplement: Supplementary file 1 [file DataSheet1.pdf]

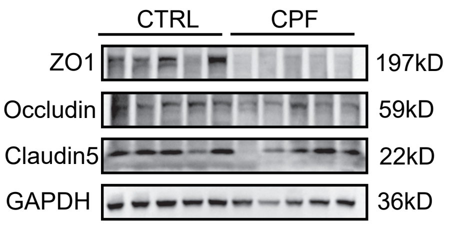

Figure 3 D in the main text

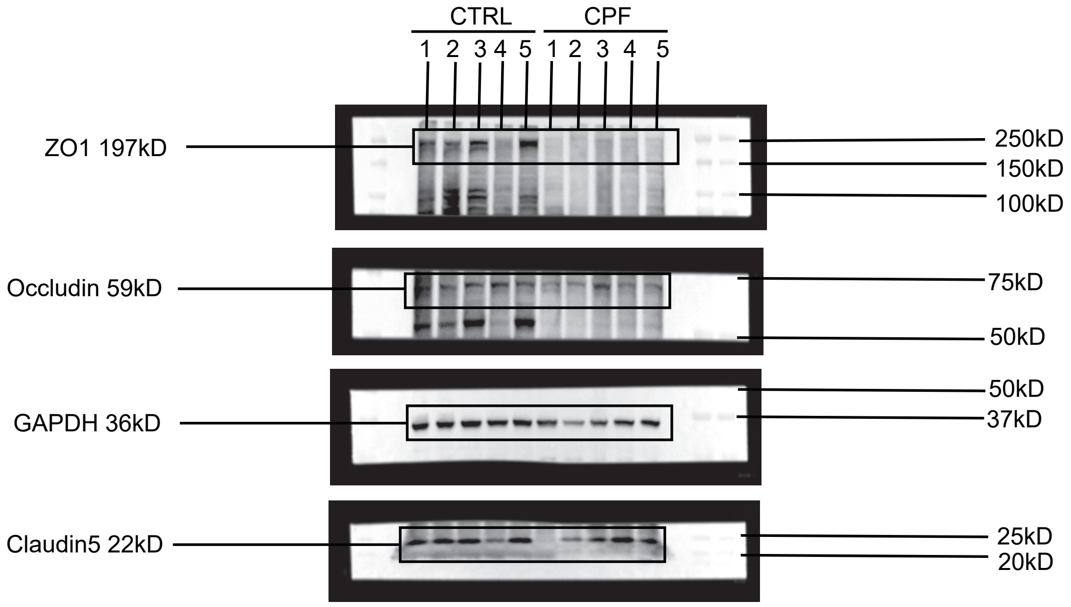

Full-length Western blot images for Figure 3 D in the main text

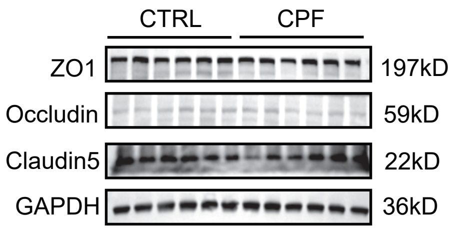

Figure 3 J in the main text

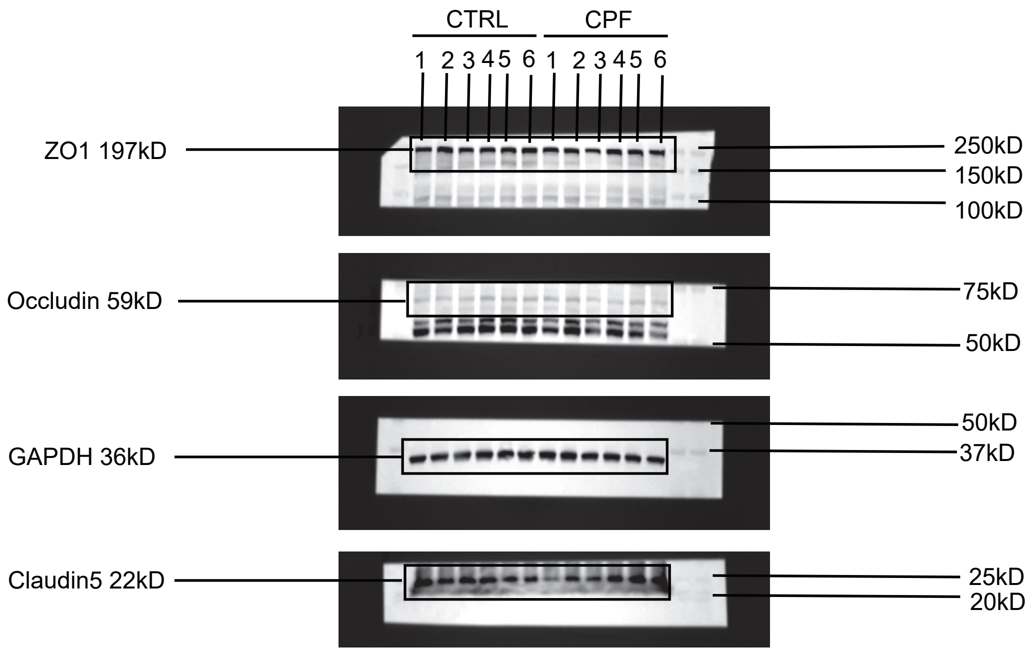

Full-length Western blot images for Figure 3 J in the main text

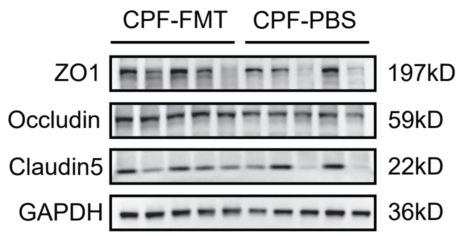

Figure 7 D in the main text

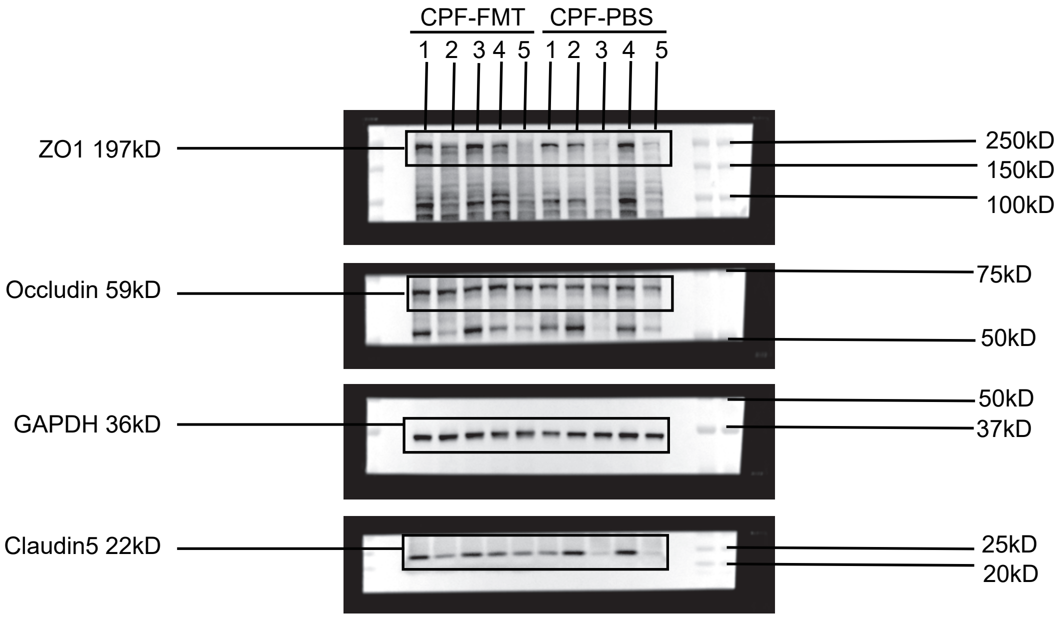

Full-length Western blot images for Figure 7 D in the main text

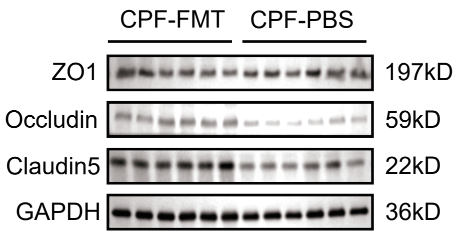

Figure 7 J in the main text

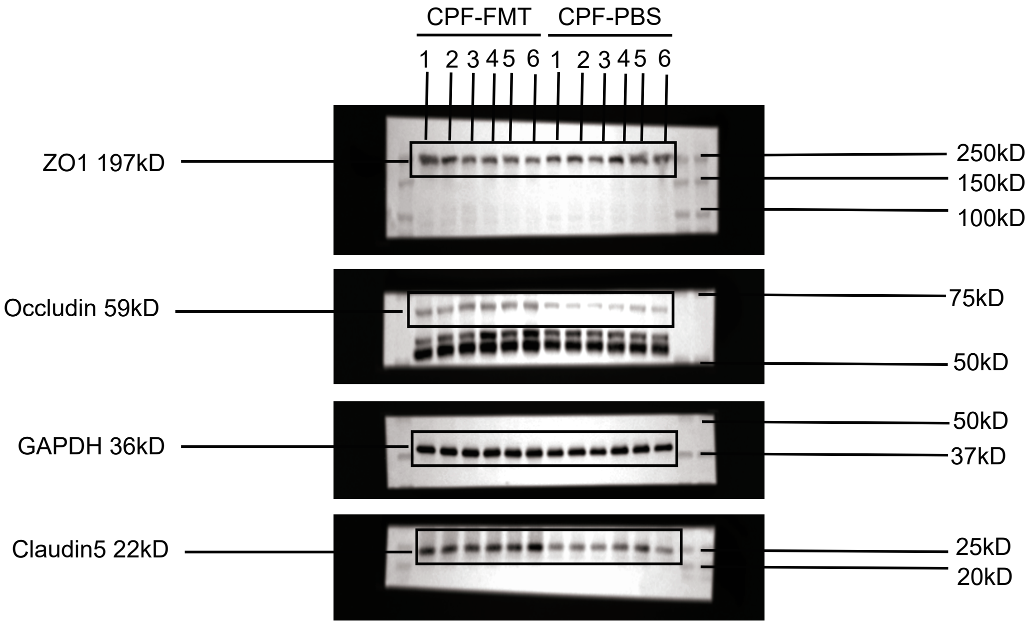

Full-length Western blot images for Figure 7 J in the main text
