# Supplementary material for: Ciprofloxacin-induced microbiota dysbiosis triggers seizure susceptibility through the microbiota-gut-brain axis
Source: Front Immunol. 2026 Mar 31;17:1670694. doi: 10.3389/fimmu.2026.1670694 (PMC13076159; doi:10.3389/fimmu.2026.1670694)
Supplement: Supplementary file 2 [file DataSheet2.docx]

**
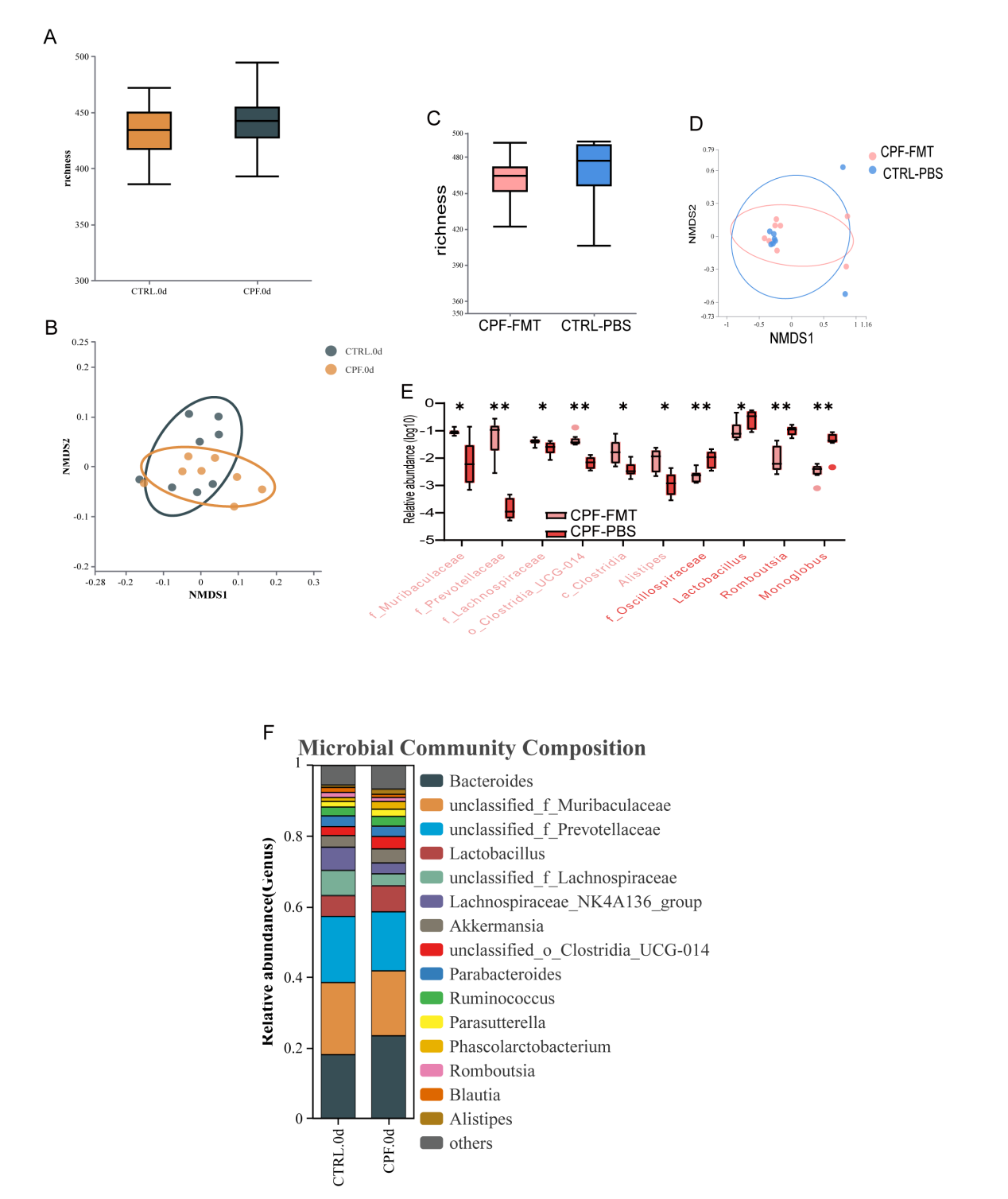
Supplemental Figure 1 Gut microbiota diversity and composition analysis at baseline and Day 28**
(A-B) Baseline gut microbiota analysis showing no significant differences in alpha diversity (richness index) and beta diversity (NMDS) between CPF.0d (n = 8) and CTRL.0d (n = 8) (P > 0.05).

1. D) By Day 28, alpha diversity and beta diversity in the CPF-FMT group (n = 8) were restored to levels comparable to the normal microbiota donor group (CTRL-PBS, n = 8) (P > 0.05).
2. Relative abundance of the top 10 differentially abundant taxa between CPF-FMT (n = 8) and CPF-PBS (n = 8). Taxa enriched in CPF-FMT and CPF-PBS are highlighted in soft red and deep red, respectively.
3. Microbial community composition at the genus level in CPF.0d (n = 8) and CTRL.0d (n = 8) at baseline, showing comparable profiles.

Data are presented as Tukey boxplots. *P < 0.05, **P < 0.01, ***P < 0.001, ****P < 0.0001.

**
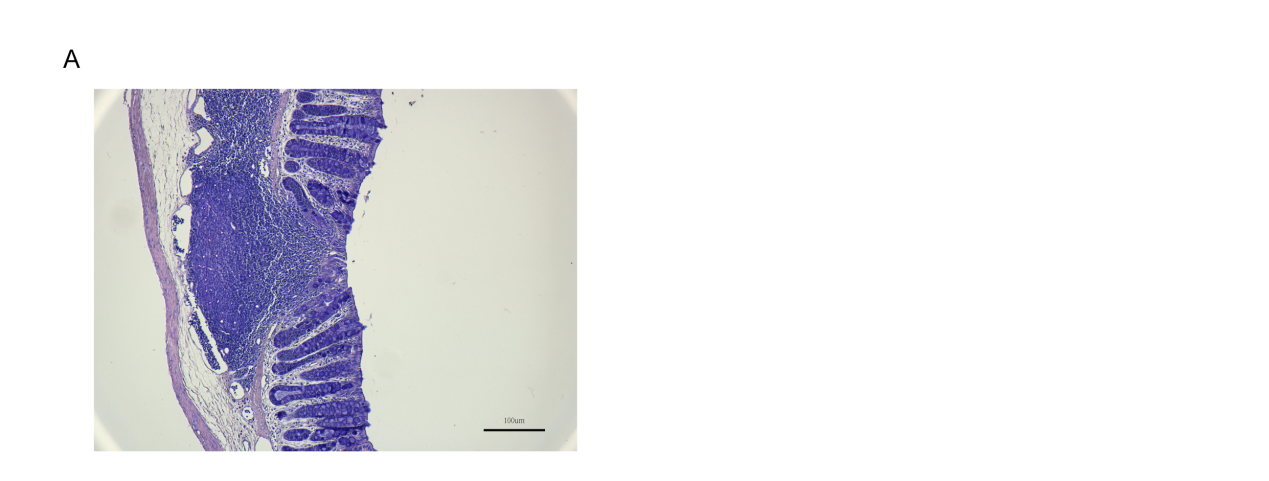
Supplemental Figure 2 HE staining of colonic tissue from the CPF group showing inflammation and villus damage**
(A) Representative histological image of colonic tissue (HE staining) from the CPF group, showing inflammatory cell infiltration in the mucosa and submucosa, as well as villus architectural damage.


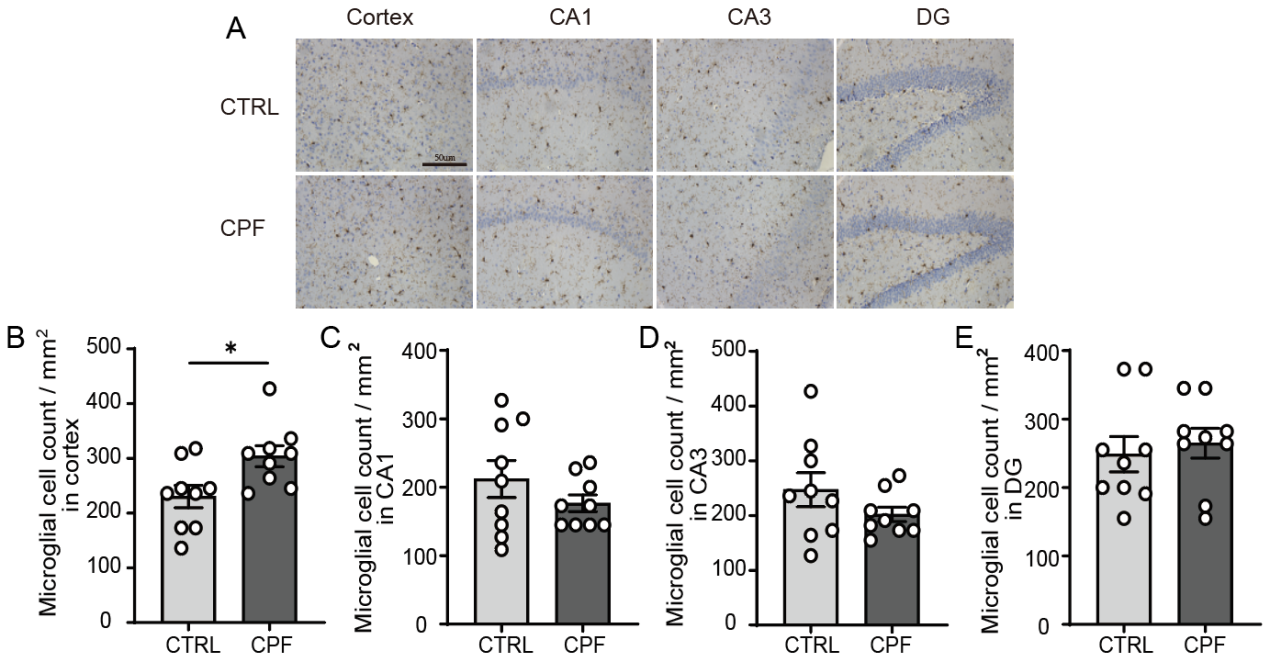
**Supplemental Figure 3 Microglial Proliferation Driven by Gut Microbiota Dysbiosis**

1. Immunohistochemical staining of Iba1 in the cortex.

(B-E) Quantification of Iba1-positive areas in the cortex, CA1, CA3, and DG regions (N = 3 per group; 3 randomly selected fields per sample).

Data are mean ± SEM; Student’s t-test was used for Significance levels: *P < 0.05, **P < 0.01, ***P < 0.001, ****P < 0.0001.


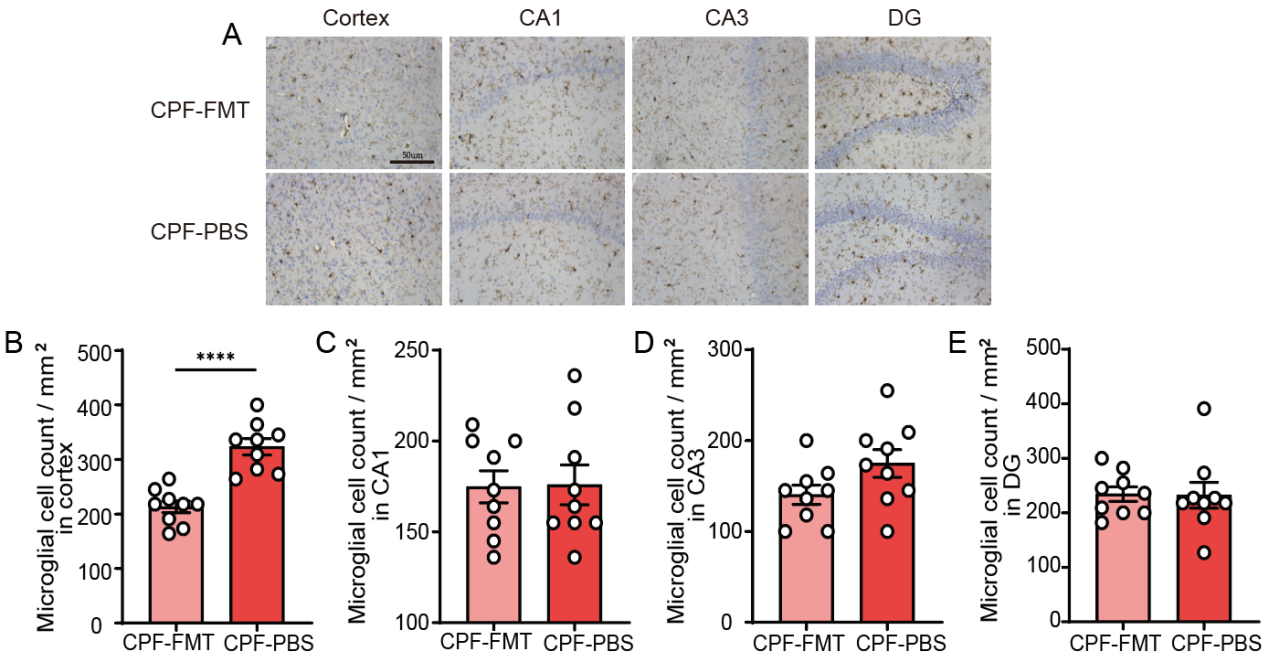
**Supplemental Figure 4 FMT Mitigates Gut Microbiota Dysbiosis-Induced Microglial Proliferation**

(A)Immunohistochemical staining of Iba1 in the cortex.

(B-E) Quantification of Iba1-positive areas in the cortex, CA1, CA3, and DG regions (N = 3 per group; 3 randomly selected fields per sample).

Data are mean ± SEM; Student’s t-test was used for Significance levels: *P < 0.05, **P < 0.01, ***P < 0.001, ****P < 0.0001.
